# Preclinical evaluation of oncolytic potential human rotavirus Wt 1-5 in gastric adenocarcinoma

**DOI:** 10.1371/journal.pone.0285543

**Published:** 2023-05-15

**Authors:** Henry Sossa-Rojas, Pedro Gabriel Franco-Maz, Carlos Zapata-Acevedo, Luz Dary Gutierrez-Castañeda, Carlos Guerrero

**Affiliations:** 1 Departamento de Ciencias Básicas y Medicina Oral, Facultad de Odontología, Universidad Nacional de Colombia, Sede Bogotá, Bogotá, D.C., Colombia; 2 Departamento de Morfología, Facultad de Medicina, Universidad Nacional de Colombia, Sede Bogotá, Bogotá, D.C., Colombia; 3 Servicio de Patología, Hospital Universitario La Samaritana, Bogotá, D.C., Colombia; 4 Departamento de Cirugía, Facultad de Medicina, Universidad Nacional de Colombia, Sede Bogotá, Bogotá, D.C., Colombia; 5 Servicio de Cirugía General, Hospital Universitario La Samaritana, Bogoté, D.C., Colombia; 6 Research Institute, Grupos Ciencias Básicas en Salud – CBS-FUCS, Fundación Universitaria de Ciencias de la Salud, Hospital Infantil Universitario de San Josá, Bogotá, D.C., Colombia; 7 Departamento de Ciencias Fisiológicas, Facultad de Medicina, Universidad Nacional de Colombia, Sede Bogotá, Bogotá, D.C., Colombia; Duke University School of Medicine, UNITED STATES

## Abstract

Despite advances in biomedical research, gastric cancer remains the leading cause of morbidity and mortality worldwide due to the limited efficacy of conventional therapies. In recent decades, oncolytic viruses have emerged as a biological therapeutic alternative to cancer due to their selectivity, effectiveness, and low toxicity. However, clinical trials have shown that developing a virus with selectivity for multiple tumor receptors and the ability to penetrate and diffuse through the tumor microenvironment to reactivate the immune system remains challenging. This study aimed to examine the oncolytic potential of tumor cell-adapted rotavirus Wt1-5 in gastric adenocarcinoma samples. This study focused on determining the propagation capacity of the RV Wt1-5 through the tumor and the importance of the expression of cell surface co-receptors, including integrin β3, protein disulfide isomerase (PDI), and heat shock proteins (Hsp-90, -70, -60, -40, and Hsc 70), during infection of tumor cells. These proteins were found to be differentially expressed in tumor cells compared to adjacent non-tumor cells. Preincubation of gastric tumor cells with antibodies against these proteins decreased rotavirus infections, validating their importance in the binding and entry of RV Wt1-5 into tumor cells, as previously reported. Upon RV infection, apoptosis was one of the types of death that was observed. This was evidenced by evaluating the expression of CASP-3, -9, PARP, cytochrome C, Bax, Bid, p53, and Bcl-2, as well as observing morphological changes such as chromatin margination, nuclear condensation, and fragmentation. Finally, at 60 h.p.i, histological analysis revealed that oncolysis compromised the entire thickness of the tumor. Therefore, the results suggest that RV Wt1-5 could be a novel therapeutic agent co-adjuvant agent for conventional and targeted therapies in managing GC. Ex vivo infection of the tumor tissue model showed characteristics of an immune response that could be explored in future studies.

## Introduction

### Gastric cancer

Gastric cancer (GC) is the fifth most common cancer and the fourth leading cause of cancer-associated mortality worldwide (GLOBOCAN 2020) [1]. GC is a heterogeneous group of tumors with variable clinical and pathological features, a high metastasis rate (25-40% in stages II-IV TNM8th—UICC/AJCC), and a 5-year relative survival rate of 30% for all combined stages of the disease and <4% for metastatic disease [2]. Surgical resection of the tumor, most commonly a radical or partial gastrectomy with an accompanying lymphadenectomy, is a fundamental part of the treatment, but is possible in less than a quarter of cases, leading to poor prognosis due to limited treatment options. Unfortunately, the current standard chemotherapy regimens for the treatment of advanced gastric cancer are not specific and have poor efficacy. They cause severe adverse reactions with poor results in terms of quality of life and patient survival [2–5]. Despite the development of new targeted therapies, such as monoclonal antibodies directed against HER2, VEGFR2, and immune checkpoint inhibitors (ICIs), the results have been far from satisfactory [6, 7].

In recent years, evidence and clinical approval have shown the potential of oncolytic viruses capable of selective replication and oncolysis, leading to the release of soluble antigens, danger signals, and cytokines, such as the type I interferons (IFNγ), tumor necrosis factor-α (TNFα) and interleukin-12 (IL-12). These cytokines drive antitumor immunity by inducing maturation of antigen-presenting cells (APCs), which, in turn, activate NK cells, CD4+ and CD8+ T cells, favoring tumor regression even in sites distant from viral infection [8–11]. A few different classes of viruses with natural or modified specificity have been explored in Phase I/IIa clinical trials as therapeutic agents for GC, such as vaccinia virus (ClinicalTrials.gov: NCT 2977156, 4226066), measles virus (ClinicalTrials.gov: NCT 4195373), adenovirus (ClinicalTrials.gov: NCT 3740256), herpes simplex virus-2 (ClinicalTrials.gov: NCT 3866525) and Newcastle disease virus (ClinicalTrials.gov: NCT 3889275), although no leading therapeutic candidate has yet emerged.

Rotavirus (RV) is the leading cause of acute gastroenteritis (AGE) in children under five years of age [12]. RV is a non-enveloped double-stranded RNA virus belonging to the *Reoviridae* family [13]. The virion is composed of three concentric capsids made up of six structural proteins (VPs) distributed as follows: outer capsid (VP7 and VP4), intermediate capsid (VP6), and inner capsid or core (VP2), which encompasses VP1, VP3 and the viral genome composed of 11 dsRNA segments. Each segment encodes a structural protein (SP) or a non-structural protein (NSP) [12, 14]. Recently, tumor-adapted rotavirus Wt1-5 was derived from the directed evolution of wild-type human rotaviruses passaged multiple times in several human cancer cell lines [15]. Different studies have shown that infection is favored by the formation of binding platforms between rotaviral proteins (VP5, VP8 and VP7) and multiple co-receptors overexpressed in tumor cells but not in non-tumor cells, such as integrins (α2β1, αVβ3, α4β1, and αxV2) and heat shock proteins (Hsp90, Hsp70, Hsp60, Hsp40, and Hsc70). These mechanisms are favored by conformational changes that depend on thiol-disulfide exchanges by protein disulfide isomerase (PDI). It has been shown that the infective capacity of RV is reduced by blocking its union with antibodies directed against these proteins [15–28].

In this study, we investigated the interaction of RV Wt1-5 with the tumor microenvironment using an ex vivo human gastric adenocarcinoma explant model for the first time. We examined the virus´s ability to infect, replicate and induce cell death in cells expressing Hsps, PDI, and integrin β3 on their outer cytoplasmic membrane. Our results showed that RV was able to spread, infect, and damage target gastric tumor tissues, leading to massive cell death within the tumor through both apoptotic and other mechanisms. Furthermore, our findings suggest that RV Wt1-5 has the potential to reactivate the immune system within tumor tissue, making it a potential candidate for virotherapy in gastric cancer.

## Materials and methods

### Cell culture

NCI-N87 [N87] CRL-5822™ human gastric cancer cells were purchased from the American Type Culture Collection (ATCC^®^ Manassas, VA, USA). Cells were cultured in RPMI 1640 medium (Sigma-Aldrich^®^, St. Louis, MO, USA) supplemented with 10% fetal bovine serum (FBS) (Invitrogen, Waltham, Massachusetts, USA), 20 U / ml penicillin, and 40 U / ml streptomycin, and incubated in a humidified atmosphere with 5% CO_2_ at 37°C.

### Histopathological and molecular characteristics of gastric cancer

#### Surgical explants

Human gastric specimens were obtained from six patients with primary gastric adenocarcinomas who underwent radical gastrectomy at the Hospital Universitario de la Samaritana (Bogotá, Colombia) between 2016 and 2019. All subjects in the study were Colombians with primary gastric cancer, preoperatively diagnosed by endoscopy, who did not receive chemotherapy before surgery and showed no signs of distant metastases. Gastric cancer tissues and corresponding non-tumor normal tissues (<5 cm) were collected. Each biopsy sample was divided into two sections: one was submitted for routine histological diagnosis, and the remaining section was used to evaluate infection with RV Wt1-5. Pathological evaluation was performed on the resected specimens after they were fixed in 10% neutral buffered formalin and sectioned into 4-mm-thick segments. The evaluation determined various characteristics of the tumor, including its diameter, location, differential degree, depth of invasion, and lymphovascular invasion. This study was approved by the Ethics Committee of Hospital Universitario de la Samaritana (approval no.2; 18 February 2016), and all experiments were conducted in compliance with the Declaration of Helsinki and guidelines for ethical principles for medical research involving human subjects. The ethics committee waived the need for consent from the participants.

#### Tissue preparation and cell viability

Human gastric cancer specimens obtained from radical gastrectomy were immediately placed in transport media RPMI 1640 supplemented with 10% fetal bovine serum (FBS), 50 IU/mL penicillin, and 50 μg/mL streptomycin after resection. The surgical specimens were washed twice with sterile phosphate-buffered saline (PBS) with pH 7.4. Tumor tissue was transferred to a 100 mm-Petri dish containing 20 mL of antibiotic-containing medium under aseptic conditions in a laminar flow hood, using sterile forceps. The tissue was then cut into ∼2 mm^3^ tissue explants using scissors and a number 22 scalpel blade, and any necrotic tissue was removed. Samples were enzymatically disaggregated after incubation with trypsin (5 mg/mL; Invitrogen, Madison, WI, USA) for 10 min at 37°C in a humidified atmosphere containing 5% CO_2_. The resulting tissue slices were further processed by washing them twice with RPMI 1640 without FBS. The tissue was then divided into portions of ∼200 mg each and placed in 50-ml centrifugation tubes (Biologix Group Ltd.). To obtain single-cell suspensions from the tumors, mechanical disaggregation was carried out using the PRO 200 Micro Homogenizer (ProScientific Inc.). The homogenizer was set at 1,000 rpm for 10 min, with RPMI without serum used as the medium. The tumor fragments and fluid underwent filtration through a Corning^®^ 100 μm Cell Strainer (Corning, NY, USA). The resulting single-cell suspension was then centrifuged at 700 x *g* for 7 min, and an aliquot was taken from each tube to determine the number of viable nucleated cells using a cell counter (Countess™ II Automated Cell Counter). For this, 10 μL of the sample was mixed with 10 μL of trypan blue (Sigma Chemical Co., St Louis, MO, USA). The cell suspension was then maintained in RPMI medium without FBS and penicillin-streptomycin.

#### Infection of disaggregated tumor cells

The tumor cell-adapted rotavirus Wt1-5 was obtained as previously described [15]. The Multiplicity of infection (MOI) was determined by analyzing the focus-forming units (FFU) through a serial dilution assay of the stock in NCI-N87 cells (ATCC^®^ CRL-5822™). The Infection was confirmed by immunocytochemistry using hyperimmune rabbit serum against RV structural proteins, and positive cells (an average between 100 and 150) were counted under an inverted microscope using a 40x objective. Finally, a viral titer of 1.2 x 10^6^ FFU/mL was determined [29].

To examine whether gastric tumor cells are susceptible to infection by RV Wt1-5, approximately 5 X 10^4^ cells were obtained from disaggregated tumors or non-tumor tissues and washed twice with RPMI 1640 medium. The cells were then inoculated with either PBS or RV Wt1-5 (MOI 0.8) activated with trypsin (10 μg/mL) (Sigma-Aldrich) in RPMI medium without FBS. The cells were incubated for 12 h at 37°C with 5% CO_2_. After incubation, the cells were fixed with 4% (vol./vol.) paraformaldehyde in PBS for 30 min at room temperature. The cells were washed twice with PBS, resuspended in PBS with 0.02% (weight/vol) sodium azide, and stored at 4°C until further assessment of tumor cell susceptibility to infection using various assays, including immunocytochemistry, epifluorescence, capture ELISA, flow cytometry and Western blot.

To determine whether viral particles with infectious capacity are generated when infecting tumor cells isolated from patients, 5x10^4^ cells/well were seeded and inoculated with RV Wt1-5 (MOI 0.8) for 1 h at 37°C with 5% CO_2_. The cells were washed twice with RPMI 1640 medium to remove the unbound viruses. After adding RPMI without FBS, the cells and supernatant were separately collected at 12 h.p.i. Three freeze-thaw cycles were performed, with cells being centrifuged at 700 x *g* during each cycle, and trypsin (10 μg/mL) was added to the supernatant for 30 min. The resulting supernatant was then inoculated into the NCI-N87 gastric cancer cell line (ATCC^®^ CRL-5822™) and incubated at 37°C with 5% CO_2_. After 12 h.p.i, cells were fixed with 4% paraformaldehyde (PFA) and analyzed by immunocytochemistry using rabbit hyperimmune serum to detect the presence of rotavirus antigens. The assay was performed three times in duplicate, with uninfected cells serving as the control.

#### Ex vivo infection of gastric adenocarcinoma

Previously reported protocols [30, 31] were used with slight modifications to examine the effects of oncolytic virus in gastric cancer explants. Briefly, human gastric adenocarcinoma samples and non-tumor gastric tissue collected by radical gastrectomy were immediately transported for processing to the Virus Molecular Biology Laboratory of Universidad Nacional de Colombia. The tissues were stored in two sterile 150 mL DURAN^®^ borosilicate glass flasks (DWK Life Sciences Tennessee, USA) with culture medium (RPMI 1640 with 10% FBS, 50 IU/mL penicillin, and 50 μg. /mL streptomycin). Under sterile conditions in the laboratory, tumor tissue was first placed in a 100 mm-Petri dish containing supplemented medium (RPMI 1640 with 10% FBS, 50 IU/ml penicillin, and 50 μg/ml streptomycin) using sterile forceps. Necrotic tissue was removed with sterile scissors and a 22-number scalpel blade, and then the tissue explants were cut into ∼ 2 mm^3^ thick sections (∼0.1g). Each explant was transferred to a new 100 mm-Petri dish with 20 mL of medium to prevent tissue desiccation. Using sterile forceps, each explant was distributed to a different well of a Corning™ 12-well flat-bottom cell culture plate containing 3 mL of RMPI 1640 medium and penicillin-streptomycin without FBS. The selected wells were inoculated with either PBS or rotavirus Wt1-5 (MOI 0.2 or 0.8) diluted in 25 μL of medium, directly onto each explant. The explants were then incubated at 37°C with 5% CO_2_ for 1 h. The unattached virus was removed by washing three times with RPMI 1640 medium. Tissue sections were harvested at 0, 12, 24, 48, and 60 h.p.i and then fixed for 1 h at room temperature with fresh 10% neutral-buffered formalin with sodium acetate or 4% (v/v) paraformaldehyde at RT, or frozen at -80°C, depending on the assay to be carried out later. The volume ratio between the sample to be fixed and fixative solution was 1:20. After fixation, three washes of at least 5 min each with PBS were performed. Cell viability was assessed at each time point, as previously described.

#### Antibodies and reagents

Primary rabbit polyclonal antibodies against rotavirus structural proteins (SPs) or non-structural proteins (NSP2-NSP6) were produced in our animal facilities. Monoclonal mouse anti-human BCL2 (catalog # IS614), mouse anti-human cytokeratin 7 (catalog # M7018), mouse anti-human carcinoembryonic antigen (catalog # GA622) mouse anti-human E-cadherin (catalog # GA059), mouse anti-human CA 19-9 (catalog # M3517), mouse anti-human CD4 (cat # M7310), mouse anti-human mutant and wild-type p53 (catalog # IS616), and polyclonal rabbit anti-human CD3 (catalog # IS503) antibodies were obtained from Dako (Agilent Technologies, Inc. Santa Clara, CA, USA). Polyclonal goat anti-human antibodies against Hsp90α/β (sc-1055), Hsp70 (sc-1060), Hsp60 (sc-1052), Hsp40 (sc-1801), Hsc70 (sc-1059), integrin β3 (sc-6626), PDI (sc-17222), and monoclonal mouse anti-human against cleaved PARP-1 (sc-56196), caspase-3 (sc-65496), caspase-9 (sc-56077), Bax (sc-20067), cytochrome c (sc-13561), Hsp90 (sc-13119), Hsp70 (sc-32239), Hsc70 (sc-7298), Hsp60 (sc-59567), Hsp40 (sc-7298), PDI (sc-376369), ERp-57 (sc-166680), integrin β3 (sc-46655), integrin β2 (sc-8420), integrin β1 (sc-374429), and monoclonal rabbit anti-human Bcl-2 (SC-783) were obtained from Santa Cruz Biotechnology Inc. (Santa Cruz, CA, USA). Monoclonal mouse antibodies against cytokeratin 8/18 (MA5-43995) were obtained from Thermo Fisher Scientific (Waltham, MA). As secondary antibodies, the following were obtained from Santa Cruz, CA, USA: mouse anti-goat IgG-PE (sc-3752), donkey anti-rabbit IgG PerCP-Cy5.5 (sc-45106), rabbit anti-goat IgG-PE (sc-3755), rabbit anti-mouse IgG-PE (sc-358926), bovine anti-goat IgG-FITC (sc-2348), mouse anti-rabbit IgG-488 (sc-516248), mouse anti-rabbit IgG-FITC (sc-2359), and donkey anti-goat or anti-rabbit antibodies conjugated with FITC (sc-362255 and sc-362261, respectively), or HRP (sc-2020 and sc-2313, respectively). 4,’6-diamidino-2-phenylindole (DAPI) was purchased from Invitrogen (Carlsbad, CA, USA).

#### Immunohistochemistry

After fixation, the tissue samples were dehydrated using an ethanol/xylene series and embedded in fresh paraffin wax at a temperature of 55–60°C. Histopathological and immunohistochemical characterization of both the tumor and non-tumor tissues were then performed for each block. The formalin-fixed paraffin-embedded (FFPE) specimen blocks were sliced into 5 μm-thick sections and subjected to immunohistochemical analyses. For the immunohistochemical staining system, a fully automatic staining device, the Ventana BenchMark XT from Ventana Medical Systems Inc. Tucson, USA (Roche Diagnostics Division) was used. The device uses a biotin-free, HRP multimer-based hydrogen peroxide substrate and 3, 3’-diaminobenzidine tetrahydrochloride (DAB) chromogen (ultraView ^TM^ Universal DAB Detection Kit, Catalog number 760-500, Ventana Medical Systems, Tucson, USA). Primary antibodies against CEA (1:100 dilution), Ck7 (1:100 dilution), E-cadherin (1:100 dilution), CA 19-9 (1:100 dilution), Bcl-2 (1:100 dilution), mutant and wild-type p53 (1:100 dilution), CD3 (1:100 dilution) or CD4 (1:100 dilution), and hyperimmune rabbit serum against rotavirus structural proteins (1:1000 v/v) were used. The slides were counterstained with hematoxylin and eosin. In addition to internal positive controls, positive control slides were included in each experiment. Antibody specificity was determined using a matched IgG isotype antibody as a negative control. The slides were examined using a Bx51 TF light microscope (Olympus Corporation) at magnification of 2x, 10x, 20x, 40x, or 60x, and image acquisition was performed using CellSens software (Olympus Corporation). Immunohistochemical reactivity was blindly interpreted by two independent investigators. For scoring assessments, cells were counted in five separate intratumoral regions under 40x magnification, and 10 representative photographs of each coverslip were taken. The percentage of antigen-positive cells was recorded using ImageJ2 2.3.0/1.53f. The area of staining was evaluated as follows: semiquantitative scoring - score 0 if < 5% of cells are positive, score 1 if 5-24% are positive, score 2 if 25-49% are positive, score 3 if 50-75%, are positive, and score 4 if > 75% are positive. Semi-quantitative scoring was also performed using IHC Profiler, with high positive, positive, low positive, and negative categories. Quantitative scoring was performed using the IHC optical density score, which ranges from 1 to 4 [32].

#### ELISA

Infection of gastric tumor cells or non-tumor cells with RV Wt1-5 (MOI 0.2 or MOI 0.8, 12 h.p.i) was assessed using the double-antibody sandwich enzyme-linked immunosorbent assay (ELISA). For this, hyperimmune sera recognizing rotavirus structural proteins (dilution 1:2000) or non-structural proteins (NSP2-NSP6) (dilution 1:2000), or monoclonal antibody 159 (nMAb159, dilution 1:2000) recognizing rotavirus structural protein VP7 [33] were used. The plates were then incubated with HRP-conjugated goat anti-rabbit IgG antibodies (0.13 μg/ml in PBS containing 1% BSA; SC-2301 Santa Cruz Biotechnology Inc.). The reaction was developed using o-phenylenediamine dihydrochloride substrate (Thermo ScientificTM Pierce^®^, Wilmington, DE, USA) in 50 mM citrate buffer (pH 5.0) containing 0.05% H_2_O_2_. The reaction was stopped by adding 2N H_2_SO_4_ and incubating for 10 min at room temperature. The plates were read using an Ultramark™ microplate reader (model 8422; Bio-Rad, Hercules, Cal, USA). The net optical density (OD) value at 490 nm was calculated by subtracting the negative control value, and the values ​​were expressed as ΔOD. To account for background signals, lysates of cells that were not infected (negative control for rotavirus detection) and incubated with all antibodies in the absence of lysates were used. Absorbance values were obtained from three independent experiments, each performed in duplicate.

#### Fluorescent IHC staining of frozen tissue sections

The procedure involved the following steps: (1) cryopreservation of fresh gastric tumor and non-tumor tissues, infected or not infected with RV Wt1-5 (MOI 0.8, 12 h.p.i). (2) Embedding and sectioning of the tissues in the Embedding Compound (OCT) and freezing at -20°C, followed by cutting tissue sections of 5 – 6 μm using a cryostat. (3) Thawing the samples and mounting them on loaded histological slides to improve tissue adhesion. (4) Drying the slides for 30 min at 37°C, followed by detection of rotavirus antigens using hyperimmune rabbit serum against rotavirus structural proteins (1:2000 vol/vol) diluted in PBS and 1% BSA for 1 h at 37°C, and then washing twice with PBS. FITC-conjugated anti-rabbit IgG secondary antibody (0.88 μg/mL Santa Cruz, CA, USA, SC 362261) diluted in 1% BSA was then incubated with 0.15 μg/mL 4’,6-diamidino-2 -phenylindole (DAPI) for 30 min at room temperature. Cells incubated in PBS-BSA without antibodies and isotype controls were used as controls. For evaluation, six representative photographs of each slide were taken using a Nikon C1 4-channel inverted confocal laser scanning microscope using the EZ-C1 software (see Gold, 3.90.).

#### Western blot

Gastric tumor cells were seeded at a density of 1 × 10^5^ cells/dish and inoculated with PBS or RV WT1-5 (MOI 0.8) for 12 h at 37° C. Twenty-five μg of total proteins from cell lysates were extracted using an SDS-loading buffer, separated by SDS-polyacrylamide gel electrophoresis (PAGE), and transferred onto a polyvinylidene difluoride (PVDF) membrane (Millipore, CA, USA) using a semi-dry transfer apparatus (Bio-Rad). After blocking with 5% skimmed milk at room temperature for 1h, the membranes were incubated with primary antibodies against specific proteins (VP7 for infection and caspases 3 and 9 for apoptosis; 0,2 μl/mL) for 1 h at 37°C and then incubated with HRP-conjugated secondary antibodies (0.13 μL/ml, Santa Cruz Biotechnology Inc., Santa Cruz, CA) for 1 h at 37°C. The membrane was washed three times, and peroxidase activity was developed using luminol reagent (Santa Cruz Biotechnology, CA, USA) according to the manufacturer’s instructions.

#### Flow cytometry analysis (FACS) and epifluorescence

The tumor cells were subjected to flow cytometry analysis to determine the expression of various proteins using, including cell surface membrane proteins such as CEA, CK-7, CK 8/18, CA 19-9, Hsp90, Hsp70, Hsp60, Hsp40, Hsc70, β1, β2, and β3 integrin; PDI and ERp57, as well as intracellular expression of rotavirus antigens and apoptotic proteins such as PARP, cytochrome C, BAX, BID, Bcl-2, caspase 3, and 9. The cells were washed twice with 50 mL of sterile PBS and centrifuged at 800 x *g* for 5 min at 4°C using a Beckman Coulter J2-MC High-Speed Centrifuge. The cells were fixed in methanol at -20°C and treated with NH_4_Cl (50 mM). The cells were then resuspended in PBS containing the primary antibody (0.2 μg/ml) in 0.1% saponin and incubated at 37°C in the dark for 1 h. After washing twice with sterile PBS, the cells were incubated with the secondary antibody (0.88 μg/mL) for 1 h at room temperature in the dark. Cells were resuspended in 300 μL of PBS for flow cytometry analysis. Uninfected cells and cells incubated without antibodies or with secondary antibodies were used as controls. The analysis was performed using a FACScanto II^TM^ flow cytometer (Becton-Dickinson, Sydney, Australia), and data were analyzed using FACSDiva^TM^ software (version 8.0, Becton-Dickinson, Franklin Lakes, NJ, USA).

For epifluorescence analysis of protein expression, the same protocol was used, except that the cells were fixed on coverslips with 4% PFA. To evaluate the images, six representative photographs were taken of each group using a 40x objective on a Nikon C1 inverted confocal laser scanning microscope. The NIS-Elements Advanced Research Program from Nikon Corp. was used to obtain the images, and the analysis was performed using ImageJ2 Version 2.3.0/1.53f. For nuclear counterstaining, 0.1 μg/mL of 4’,6-diamidino-2-phenylindole (DAPI) was added for 30 min in a humid chamber at 37°C, protected from light.

#### Isolation of cell membrane‐enriched fractions

Cell membrane-enriched fractions were isolated according to a previously described method (Lin et al., 1987) [34]. Briefly, tumor cells isolated from patients with gastric subtype-intestinal adenocarcinoma (Ti2) (1 × 10^8^) were washed with PBS and treated with a hypotonic solution (5 mM HEPES, pH 7.4-, and 50-mM sucrose) for 5 min. The cells were then lysed using a glass homogenizer (DWK Life Sciences Tenbroeck, 40 ml, Fisher Scientific ^®^, USA) on ice. Cell membrane aggregates were sedimented by adding CaCl_2_ (final concentration 10 mM) and centrifuging the mixture at 5,000 x *g* for 15 min at 4°C. The supernatant was further centrifuged at 20,000 x *g* for 30 min. The supernatant was discarded, and the pellet was resuspended in sterile PBS and stored at 4°C for up to 24 h until use.

#### Binding assay

The binding of rotavirus particles to tumor cells was characterized by treating tumor cell membrane-enriched fractions with goat anti-human polyclonal antibodies (0.2 μg/mL Santa Cruz, CA, USA) against Hsp90, Hsp70, Hsp60, Hsp40, Hsc70, integrin β3, and PDI. Tumor cell membrane-enriched fractions were incubated with RV Wt1-5 (MOI 0.8) overnight at 4°C, followed by solubilization with RIPA buffer (50 mM Tris–HCl, pH 8.0, 150 mM NaCl, 1% NP-40, 0,5% DOC, and 0.1% SDS) and 1mM protease inhibitor phenylmethanesulfonylfluoride (PMSF) for 30 min at 37°C. RIPA buffer-solubilized cell membrane-enriched fractions were added to guinea pig hyperimmune serum-coated ELISA plates as capture antibodies (1:1000) to rotavirus structural proteins in duplicate and incubated overnight at 4°C. After three washes with washing buffer (PBS with 0.05% Tween 20 [PBS-T]), non-specific binding sites were blocked by incubation with 5% skimmed milk in PBS-T for 1 h at 37°C. Next, goat primary antibodies (0.2 μg/mL Santa Cruz, CA, USA) against Hsp90 (SC-1055), Hsp70 (SC-1060), Hsp60 (SC-1052), Hsp40 (SC-1801), Hsc70 (SC-1059), integrin β3 (SC-6626), or PDI (SC-17222) were added in PBS containing 1% BSA and incubated for 1 h at 37°C. After three washes with PBS-T, secondary HRP-conjugated donkey anti-goat IgG antibodies (0.133 μg/mL, SC-2020) were added and incubated for 1 h at 37°C. After washing three times with PBS-T, the reaction was developed using o-phenylenediamine (OPD) and H_2_O_2_, and then stopped by adding 2 N H_2_SO_4_. The plates were read using an Ultramark™ Microplate Reader (model 8422, Bio-Rad). The net value of optical density (OD) at 490 nm was obtained after subtraction of the negative control value, and values were expressed as ΔOD. The assays were performed three times in duplicate.

#### Antibody blocking assay

The tumor cell membrane-enriched fractions were incubated separately for 1 h at 37°C with rabbit F(ab’)2 fragments against Hsp90, Hsp70, Hsp60, or Hsp40 (0,04 mg/ml), or with antibodies against Hsc70 (SC-1059), integrin β3 (SC-6626), or PDI (SC-17222) (4 μg/ml). The F(ab’)2 fragments were prepared using a solid-phase method as previously described [35]. The peptide sequences used to induce the antibodies were as follows:

Hsp90 (620-RDNSTMGYMAAKKHLEINPDHS-641)

Hsp70 (705-QIQQYMKIISSFKNKEDQYDHLD-727)

Hsp70 (646-NSFTLKLEDTENWLYEDGDQPKQ-668)

Hsp70 (741-AMEWMNNKLNLQNKQSLTMDP-761)

Hsp60 (393-RLAKLSDGVAVLKVGGTSDVEVN-415)

Hsp40 (251-GSDVIYPARISLREALCGCTVNV-273).

Excess F(ab’)2 fragments and antibodies were removed by diluting the fractions with 1 mL of ice-cold PBS and centrifuging at 650 x *g* for 5 min. The supernatant was then removed, and the precipitate was incubated with RV Wt1-5 (MOI 0.8), as described above. Tumor cell membrane-enriched fractions without F(ab’)2 fragment pre-treatment and incubated with RV Wt1-5, as well as tumor cell membrane-enriched fractions inoculated with ovalbumin instead of the virus, were used as controls.

#### In vitro RV Wt 1-5 infectivity and antibody blocking assay

The role of cell surface proteins PDI, integrin β3, and Hsps in rotavirus infection was tested by treating NCI-N87 cells with antibodies against these cellular proteins. Cells (1×10^5^ cells/100 μl of RPMI without FBS) were separately incubated with different dilutions (0.025, 0.0125, and 0.0031 μg/mL) of F(ab’)2 fragments against Hsp90, Hsp70, Hsp60 or Hsp40, or antibodies to Hsc70 (SC-1059), integrin β3 (SC-6626), or PDI (SC-17222) in RPMI 1640 containing 1% BSA, for 1 h at 37°C. The cells were then washed twice with RPMI 1640 to remove unbound antibodies, incubated on ice for 5 min, and immediately inoculated with 100 μL of ice-cold RPMI 1640 containing RV Wt1-5 at MOI 0.8. The inoculated cells were then incubated for 45 min at 4°C, washed with RPMI 1640 to remove the unbound virus, and incubated in RPMI without FBS for 12 h at 37°C in a 5% CO2 environment. After incubation, the cells were fixed with 4% PFH for 30 min at room temperature, washed twice with PBS, and processed for an immunochemistry assay to determine the percentage of infected cells, as indicated above. Cells without antibody pretreatment and incubated with rotavirus, as well as cells inoculated with ovalbumin instead of the virus, were used as controls.

#### Statistical analysis

Statistical analysis was performed for multiple comparisons using Mann-Whitney U, Kruskal-Wallis, and two-way analysis of variance (ANOVA) with Dunnett´s multiple comparison tests. The data were presented as mean ± standard deviation (SD) from three biological replicates, and p-values less than 0.05 or 0.0001 were considered statistically significant. GraphPad Prism V.8 (GraphPad Software, La Jolla, California, USA) and Stata 12 software (StataCorp, Texas, USA) were used to calculus statistical values. Significance was indicated with an asterisk.

## Results

### Histopathologic evaluation of tumor tissue

In this study, surgical specimens obtained after total gastrectomy from six patients diagnosed with gastric adenocarcinoma by the Surgery Service of the Hospital Universitario de La Samaritana (HUS) were analyzed. All clinical and biological data of the specimens were available (Table 1).

**Table 1:** Patient clinical and histopathological data.

Patient	Gender	Age (years)	Tumor Size (cm)	Tumor location	Differential degree	Lauren classification	pTNM stage AJCC/UICC 8th	Treatment
**1**	F	55	>5	Pyloric antrum	Poor	Difuse	III B T3N1M0	Radical gastrectomy
**2**	M	64	>5	Pyloric antrum	Moderate	Intestinal	III B T3N1M0	Radical gastrectomy
**3**	M	66	>5	Pyloric antrum	Poor	Difuse	III B T3N1M0	Radical gastrectomy
**4**	M	54	>5	Pyloric antrum	Poor	Difuse	III B T3N1M0	Radical gastrectomy
**5**	M	65	>5	Greater curvature	Moderate	Intestinal	III B T3N1M0	Radical gastrectomy
**6**	M	48	>5	Pyloric antrum	Poor	Difuse	III B T3N1M0	Radical gastrectomy

The predominant histological subtype of the tumors analyzed was diffuse-subtype gastric adenocarcinoma (66.7%), followed by intestinal-subtype gastric adenocarcinoma (33.3%). The mean age of patients with diffuse-subtype was 55.7 years, and three of these patients (75%) were men. The mean age of patients with the intestinal-subtype was 65 years, and both of these patients (100%) were men. Anatomically, five cases were located in the distal region of the stomach in the pyloric antrum (83.3%), and one case was located in the proximal region (16.7%). All tumors were greater than 5 cm in diameter and had invaded the serosa and lymph nodes. The expression of gastric tumor markers in surgical samples of tumor and non-tumor tissues was evaluated using immunocytochemistry and flow cytometry.

**Fig 1:**
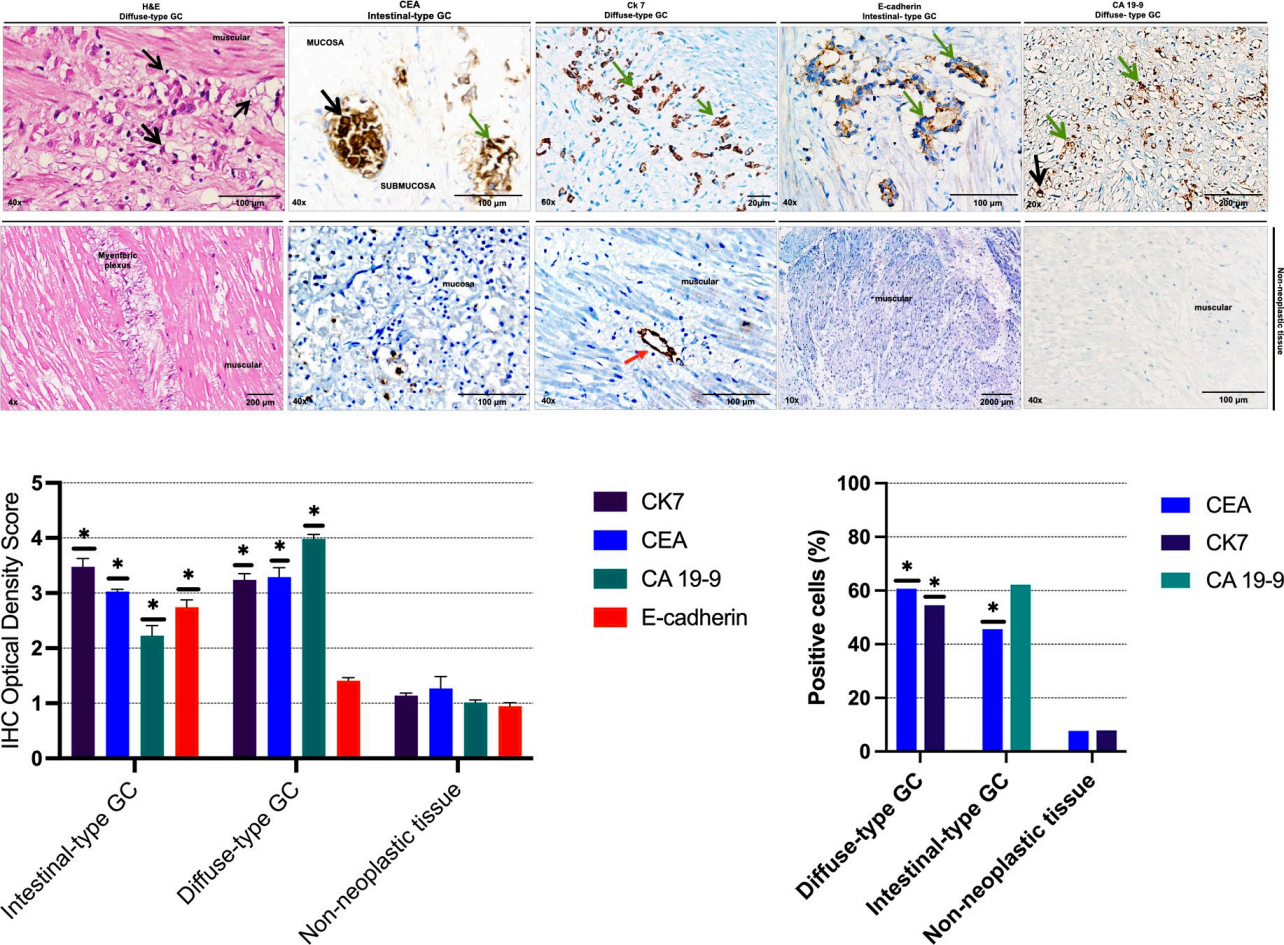
**Expression of tumor markers in tissue samples from patients diagnosed with gastric adenocarcinoma. (A)** Representative images of hematoxylin and eosin (H&E) staining and immunohistochemical staining (CEA, CK-7, E-cadherin, CA 19-9) in gastric adenocarcinoma and non-tumor tissues. Black arrows show signet-ring neoplastic cells, green arrows show tumor cell aggregates, and red arrows show endothelial cells. **(B)** Quantification of immunohistochemical analysis by the IHC Optical Density Score method in tumor tissue and non-tumor tissue samples. Data are shown as mean ± SD. Statistical significance is indicated by p-values (* p < 0.05). **(C)** Flow cytometric evaluation of tumor marker expression in cells isolated from gastric adenocarcinoma and non-tumor tissues.

The expression of gastric tumor markers in surgical samples of tumor and non-tumor tissues was evaluated using immunocytochemistry and flow cytometry. The expression of gastric tumor markers CEA, Ck-7, E-cadherin, and CA 19-9 was evaluated by immunohistochemistry (Fig 1A and 1B) and flow cytometry (Fig 1C) in explant tissues of diffuse and intestinal-subtype gastric adenocarcinomas and in adjacent non-tumor gastric tissues, encompassing the different layers of the stomach wall, including mucosa, submucosa, and muscularis propria. As shown in Fig 1B, the IHC Optical Density Score for Quantitative Immunohistochemistry Image Analysis in the group of intestinal-subtype malignant lesions of the gastric wall were 3.35 (Ck7), 2.98 (CEA), 2.08 (CA 19-9), 2.61 (E-cadherin); and in the diffuse-subtype, they were 3.13, 3.10, 3.99, and 1.34, respectively. In contrast, the values in adjacent non-tumor tissues were 1.13, 1.28, 1.066, and 1, respectively. These values were significantly higher in the gastric cancer group than in the non-tumor tissue group (p*<0.05). Similarly, the expression percentages of gastric cancer tumor markers evaluated by flow cytometry were higher for diffuse-subtype GC, 60% (CEA), and 54.5% (Ck7), and for the intestinal-subtype, they were 45.6% (CEA) and 62.2% (CA 19-9), when compared with the non-neoplastic surrounding tissue 7.7% (CEA) and 7.9% (Ck 7) (Fig 1C).

### Rotavirus infection of gastric adenocarcinoma

**Fig 2:**
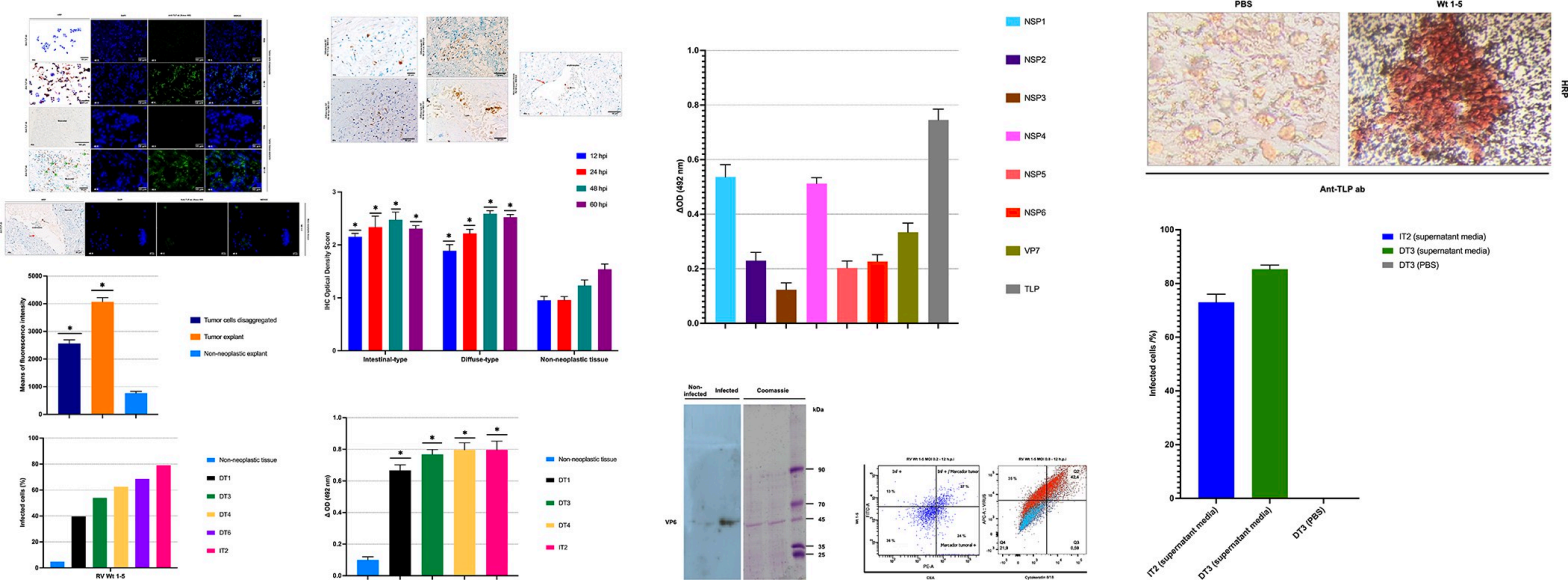
Infection of gastric cancer tumor cells by RV Wt 1-5. Gastric tumor cells and explants were infected separately with RV Wt1-5 (MOI 0.8 or MOI 0.2) or mock-inoculated with PBS. **(A)** Representative images of HRP immunochemistry staining of rotavirus structural antigens and Alexa 488 fluorescent staining (green) at 12 h.p.i, MOI 0.8 after infection in tumor cells disaggregate, tumor explant and **(B)** non-neoplastic tissue explants. **(C)** Mean Fluorescence Intensity of the cell population infected with Wt1-5 (MOI 0.8) in comparison with the control cells at 12 h.p.i. **(D)** Quantification of RV antigens in isolated gastric adenocarcinoma cells from different tumors using flow cytometry at MOI of 0.2 (dark bar) and MOI of 0.8 (color bars) in comparison with non-tumor tissue control at 12 h.p.i. DT, diffuse-subtype gastric adenocarcinoma; IT, intestinal-subtype gastric adenocarcinoma. **(E)** Representative images of the immunochemistry assay for viral structural antigens from gastric tumor and non-neoplastic tissue explants inoculated with RV Wt1-5 at different times of infection (12, 24, 48, and 60 h.p.i). **(F)** HRP immunochemistry staining of RV structural antigens at 12, 24, 48, and 60 h.p.i was quantified according to the IHC optical density score as the number of cells positive for rotavirus structural antigens using HRP immunochemistry assay. **(G)** Evaluation of rotavirus antigens in RV-infected gastric cancer and non-neoplastic tissue at an MOI of 0.2 (black bar) or MOI of 0.8 (color bars) at 12 h.p.i by Capture ELISA. **(H)** As in G, except that cells positive for non-structural proteins NSP2, NSP3, NSP4, NSP5, NSP6, structural antigen VP7 (Ab159), and rotavirus structural antigens were recorded. **(I)** Western blot evaluation of the RV structural protein VP6 in gastric tumor cells previously infected with RV Wt1-5 (MOI 0.8) for 12 h.p.i. **(J)** Analysis by flow cytometry of infection by RV Wt 1-5 (MOI 0.2 or 0.8) of tumor cells expressing tumor markers CEA and cytokeratin 8/18 at 12 h.p.i. **(K)** Representative image of IHC evaluation of infection of NCI-N87 cell line. Tumor explants were inoculated with RV Wt1-5 (MOI 0.8) or with PBS, and at 12 h.p.i the supernatant was collected, centrifuged, and then used to inoculate the NCI-N87 cell line. The presence of rotavirus antigens was evaluated using hyperimmune sera against RV. **(L)** The quantification is shown in the bar chart. (IT2 intestinal-subtype GC, DT3 diffuse-subtype GC). Data from three independent experiments performed in duplicate are shown as mean percentages ± standard deviation (SD). Mann-Whitney U test with a value *p<0.05.

To explore the capacity of RV Wt1-5 to replicate in gastric adenocarcinoma tumor cells, two infection models were established: infection of disaggregated tumor cells and ex vivo infection of tumor explants. RV Wt1-5 was derived from human parental rotavirus as previously described [15]. Tumor tissue explants and tumor cells obtained from six patients who underwent radical gastrectomy were inoculated with PBS (mock) or infected with RV Wt1-5 (MOI 0.8 and 0.2). Infected non-neoplastic gastric tissue, isolated from the area distal to the tumor location, was used as a control. Immunohistochemical and epifluorescent immunolocalization of rotavirus structural antigens showed increased expression in gastric tumor cells and explants compared to non-neoplastic tissues (Fig 2A and 2B), with all tumors exhibiting high-intensity staining while normal tissues did not (Fig 2C). Flow cytometry data analysis revealed that all tumors in this study were susceptible to RV Wt1-5 infection, with infection percentages ranging between 40% and 79% (MOI 0.2 and 0.8) at 12 h.p.i. which was more efficient compared to non-tumor gastric tissue (Fig 2D). It is noteworthy that the infection percentages in gastric tumors evaluated were higher when MOI increased. Moreover, IHC imaging of tumor infection demostrated large areas of viral spread and infection throughout the tumor, with necrosis of neoplastic tissue evident at 48 and 60 h.p.i. The adjacent normal tissues were negative for infection and tissue lysis (Fig 2E). Similarly, the IHC optical density score indicated a significant increase in tumor infection from 12 to 60 h.p.i compared to adjacent non-neoplastic tissue (p*>0.05) (Fig 2F). Therefore, the tumor microenvironment does not compromise RV replication and spread within the tumor. It is important to note that IHC Optical Density Score values in non-tumor tissue increased after 48 h due to the presence of rotaviral antigens in the cytoplasm of immune cells.

Capture-ELISA revealed the presence of viral antigens in the evaluated tumors after 12 h.p.i at 37°C (Fig 2G). The results of capture ELISA for non-structural proteins (Fig 2H) and WB (Fig 2I) indicate that the RV is replicating in the tumor cell. Infected cells are shown to be gastric epithelial cells by correlating infection with CEA and cytokeratin 8-18 markers expression. Consistent with previous results, an increasing proportion of cells positive for rotavirus antigens is evident, depending on MOI increase (Fig 2J). Infection of tumor cells and subsequent release of virions that spread through the tumor, infecting adjacent cells in the tumor microenvironment (TME), pose some challenges for oncolytic viral therapy. To evaluate whether inoculation of gastric tumor cells with RV Wt1-5 generates virions with infectious capacity in the medium, we used the supernatant obtained at 12 h.p.i from two explants (TI2, TD3) inoculated with RV Wt1-5 (MOI 0.8) to infect the gastric cancer cell line NCI-[N87] (ATCC^®^ CRL-5822™). Immunohistochemical staining results showed infection percentages of 76% (intestinal-subtype GC 2) and 84% (diffuse-subtype GC 3), indicating that the tumor supernatant contained mature virions with infective capacity. This result validates the rotavirus replicative cycle reported in the literature between 8 and 10 h.p.i and the ability to release infectious virions into the environment at 12 h.p.i [36] (Fig 2K and 2L).

Our findings indicate that both intestinal and diffuse-subtype gastric adenocarcinomas are susceptible to RV Wt1-5 infections and that RV efficiently infects these tumors. These results suggest that gastric tumor cells are permissive to infection by RV Wt 1-5, while normal tissues are not.

### Expression of cell surface proteins in gastric adenocarcinoma

The success of oncolytic viral therapy depends on the virus`s ability to selectively target tumor cells, which are more susceptible to infection due to the overexpression of a repertoire of cell surface receptors that facilitate virus attachment and entry. This selectivity enables viral genes to be expressed, resulting in replication and subsequent lysis of the tumor cell [37]. Several cell surface proteins have been suggested to be involved in rotavirus entry into the host cell, such as Hsc70, integrins (αVβ3, α2β1, α4β1, and αxβ2), and protein disulfide isomerase (PDI) [17, 20, 22, 24, 25, 27, 35, 38–40].

**Fig 3:**
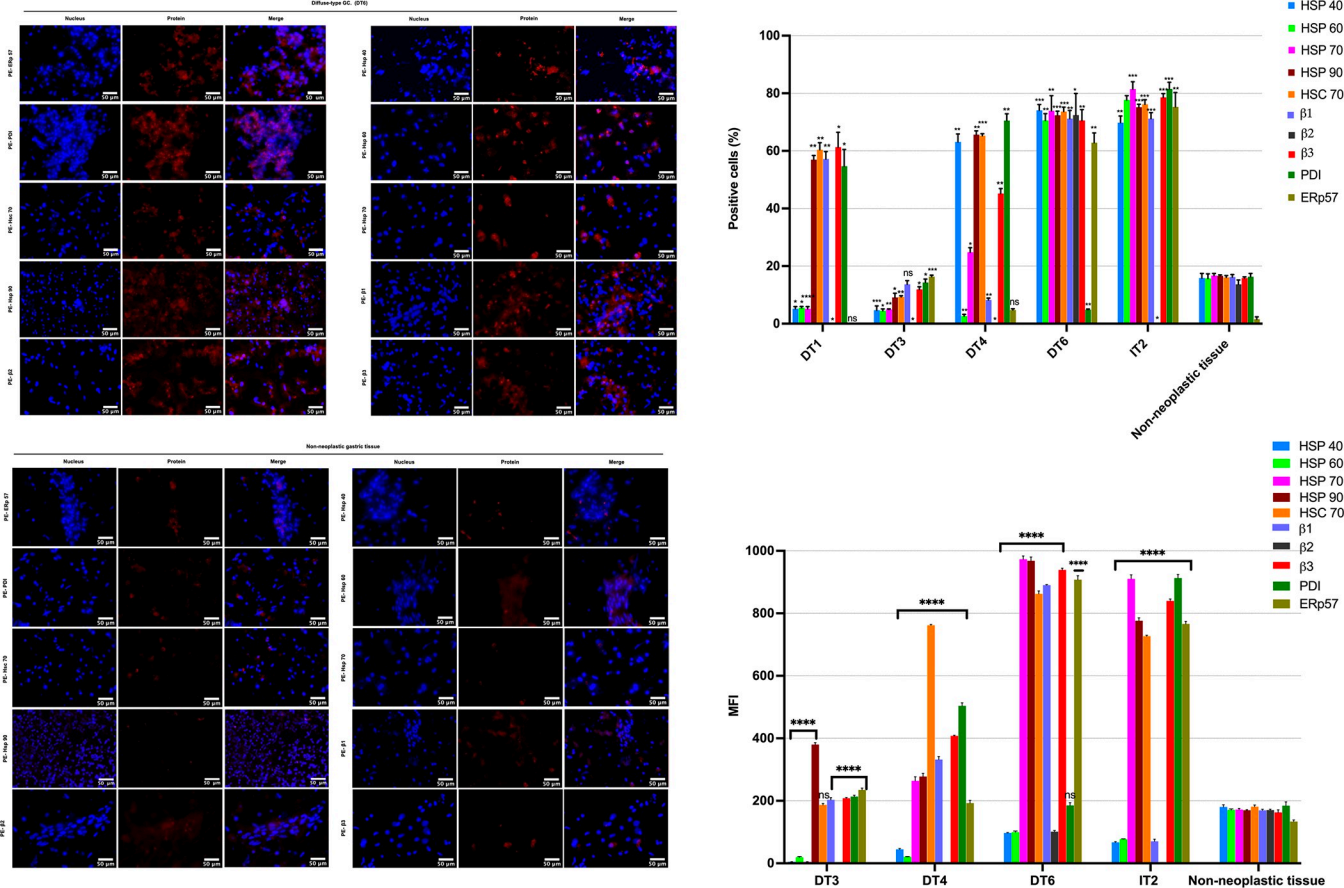
Cell-surface protein expression in gastric adenocarcinoma and non-neoplastic adjacent tissues. **(A)** Representative immunofluorescence staining of membrane proteins in gastric tumors and **(B)** adjacent non-neoplastic gastric tissues. Membrane protein expression was observed at a higher frequency and intensity in gastric tumors. Magnification: ×40. Scale bar: 50 μm. **(C)** Expression levels of proteins on the cellular membrane of gastric cancer cells and non-tumor cells were measured using flow cytometry. DT: diffuse-subtype gastric adenocarcinoma; IT: intestinal-subtype adenocarcinoma. **(D)** The median fluorescence intensity (MFI) was determined using flow cytometry to evaluate the expression of membrane proteins, as described in (C). Data from three independent experiments performed in duplicate are shown as mean percentages ± standard deviation (SD). Two-way ANOVA test with a value p<0.0001. (****p<0.0001, ***p<0.001, **p<0.01, *p<0.1).

To investigate the presence of these proteins on the cell surface of gastric cancer cells, we used antibodies against Hsp40, Hsp60, Hsp70, Hsp90, Hsc70, PDI, ERp57 and integrins β1, β2, and β3 for epifluorescence (Fig 3A and 3B) and flow cytometry analysis (Fig 3C).

The flow cytometry analysis showed that gastric tumors were positive for Hsp40 (0%, 65.3%, 72.4%, 74.3%), Hsp60 (2.4%, 4.7%, 72.7%, 76%), Hsp70 (0%, 25.4%, 75.8%, 81.5%), Hsp90 (11.2%, 33.7%, 57.6%, 74.4%, 77.3%), Hsc70 (9.7%, 58.2%, 64.6%, 72.4%, 75.5%), integrin β1 (8.7%, 13.6%, 56.6%, 71.7%, 73.2%), integrin β2 (78.3%), integrin β3 (12.8%, 43.6%, 59.9%, 72.5%, 77.4%), PDI (0, 14.8%, 57.7%, 68.3%, 81.2%), and ERp57 (0%, 16.6%, 62.5%, 78%). In contrast, non-tumor tissue showed lower percentages of expression of Hsp40 (16.5%), Hsp60 (16.2%), Hsp70 (16.6%), Hsp90 (16.3%), Hsc70 (16.7%), integrin β1 (16.4%), integrin β2 (14%), integrin β3 (16.2%), PDI (17.1%), and ERp57 (1.5%) (Fig 3C). Variable changes in the median fluorescence intensity (MFI) were also observed for the proteins tested in tumor cells compared to non-tumor cells. Notably, the diffuse-subtype 3 tumor showed the lowest intensity values in expression (Fig 3D).

### Binding of rotavirus to Hsps, PDI, and integrin β3

**Fig 4:**
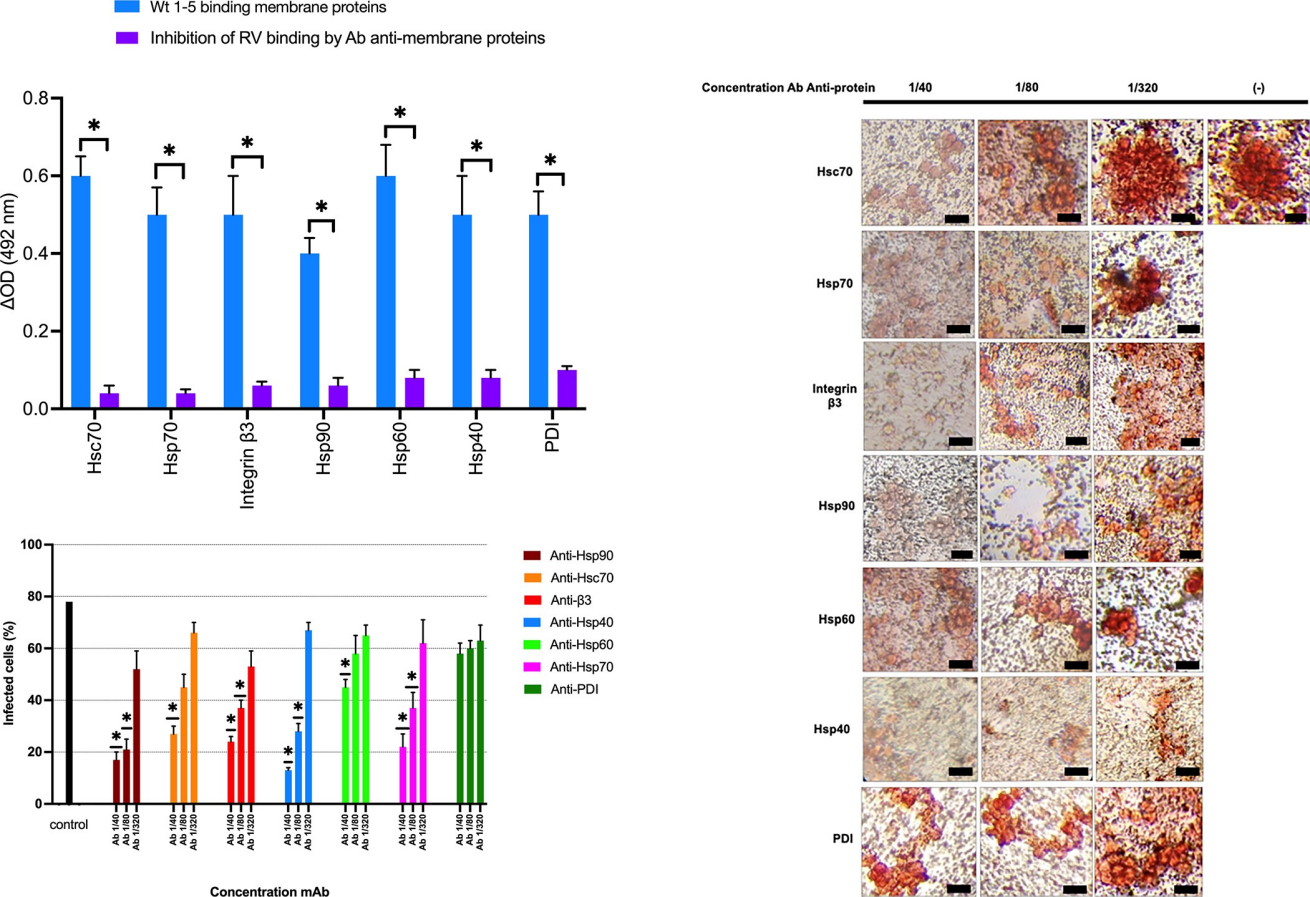
Binding of rotavirus particles to Hsp90, Hsp70, Hsp60, Hsp40, Hsc70, PDI, and integrin β3 and blocking of RV binding to the tumor cell membrane-enriched fractions surface by antibodies directed to these proteins. **A.** Tumor cell membrane-enriched fractions were incubated with F(ab)2` fragments against synthetic peptides derived from Hsp90, Hsp70, Hsc70, Hsp60, Hsp40, or mAbs against β3 or PDI, followed by incubation with RV Wt1-5 (MOI 0.8). After incubation, were solubilized in RIPA buffer and captured in ELISA plates coated with guinea pig-generated capture antibodies against rotavirus structural proteins (TLPs). Goat polyclonal primary antibodies against Hsps, β3 integrin, and PDI were used for detection. The reaction was measured using an HRP-conjugated donkey anti-goat IgG secondary antibody and developed using an OPD substrate. Fractions that were unblocked with antibodies were used as binding controls. **B**. Inhibition of rotavirus infection by antibodies against Hsp90, Hsp70, Hsp60, Hsp40, Hsc70, β3 integrin, or PDI. NCI-N87 cells were separately incubated with the indicated dilutions of the F(ab)`2 fractions of antibodies against the different proteins for 1 h at 37°C. After incubation, cells were washed with RPMI and incubated with RV WT1-5 for 45 min at 4°C and then washed with medium and incubated in RPMI without FBS for 12 h at 37°C and 5% CO2. Rotavirus structural antigens were determined by immunocytochemistry. Cells without antibody pretreatment and incubated with the RV and cells without rotavirus and without antibody were used as controls. Data are presented as the percentage of infected cells. **C.** Representative images of the previous immunocytochemistry for RV Wt1-5 (Scale bar = 50 μm). Data of three independent experiments performed in duplicate are shown as mean percentages ± standard deviation (SD). The Mann-Whitney U-test was used, with a value of p<0.05.

To determine whether RV Wt1-5 binds to co-receptor cell surface proteins (Hsp90, Hsp70, Hsp60, Hsp40, Hsc70, integrin β3, or PDI) during the initial events of infection, enriched membrane fractions obtained from gastric subtype-intestinal adenocarcinoma (Ti2) were incubated with RV in ELISA plates with hyperimmune sera against RV as capture antibody, and primary antibodies were used for the detection of each of the cellular proteins evaluated. As a control, membranes were incubated with rabbit F(ab’)2 fragments against synthetic peptides derived from Hsp90, Hsp70, Hsp60, Hsp40, or antibodies against integrin β3 (SC-6626) or PDI (SC-17222) prior to inoculation with RV Wt1-5. After revealing the reaction, ΔOD values were significantly reduced for all proteins tested by rotavirus binding blockade compared to fractions without pretreatment with F(ab’)2 fragments (p<0.05) (Fig 4A). These findings suggest that rotavirus particles can stably interact with Hsp90, Hsp70, Hsp60, Hsp40, Hsc70, PDI, or β3 integrin, using the amino acids that the F(ab`) fragments recognize or nearby areas (specifically of the Hsps) that are overexpressed in the cytoplasmic membrane of gastric tumor cells.

### Blocking of viral infection with anti-HSPs, PDI, and β3 antibodies

To further confirm the participation of cytoplasmic membrane proteins hsp90, hsp70, hsc70, hsp60, hsp40, β3 integrin, and PDI during RV infection, a blocking assay was performed on the gastric cancer cell line NCI-N87. Different dilutions of the F(ab)`2 fragments of the antibodies that recognize only 20 amino acids of each Hsp protein (as this area or nearby regions participate in the binding and infection of the virus) and mAbs against β3 integrin or PDI were separately incubated. Immunocytochemical analysis revealed that when the F(ab)`2 portions of the antibodies against each cell surface protein were used at the highest concentration (0.025 g/mL), Wt1-5 infection was reduced by 61-20% (p<0.05) (Fig 4B). The proteins associated with a greater percentage decrease in infection were, in descending order, Hsp40 (61%), Hsp90 (61%), Hsp70 (56%), β3 (54%), Hsc70 (51%), Hsp60 (33%), and PDI (20%) (Fig 4B and 4C). These results demonstrate the heterogeneity in the participation of co-receptor proteins during the early interactions of RV Wt1-5, which promote the infection of gastric tumor cells. These mechanisms are likely facilitated by the aberrant expression of these proteins in the cytoplasmic membrane of neoplastic cells.

### Genotoxic and apoptotic effects in gastric adenocarcinoma cells inoculated with the RV Wt1-5

**Fig 5:**
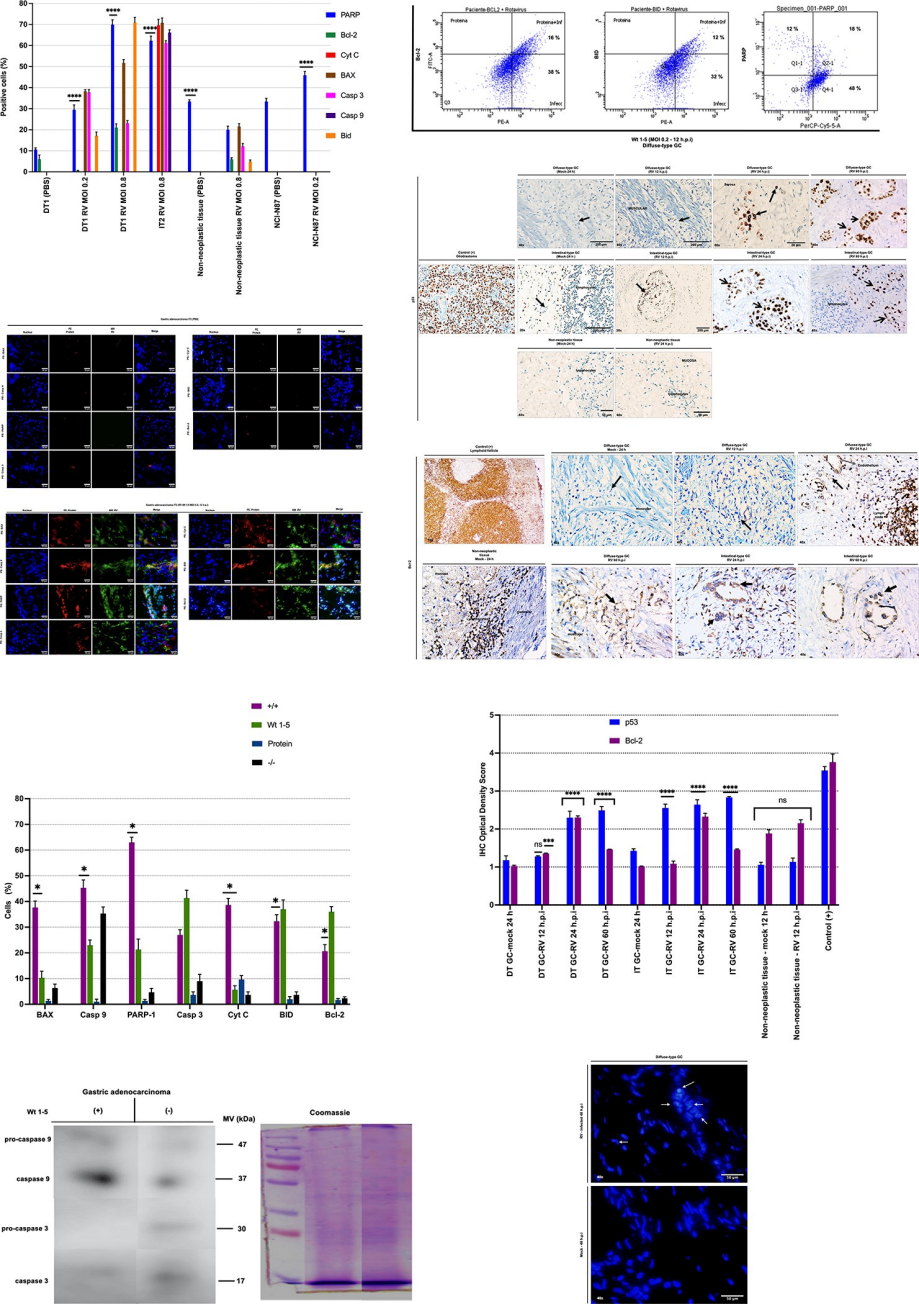
Genotoxic and apoptotic effects induced by Wt1-5 rotavirus infection. Gastric tumors were infected with Wt1-5 (MOI 0.2 or 0.8) for 12 h.p.i. and non-infected tumor tissue and non-tumor tissue were used as controls. (A) Flow cytometry was used to evaluate the expression of PARP, Bcl-2, Cyt C, BAX, caspase 3, caspase 9, and BID. The percentages of cells showing the expression of apoptotic proteins and DNA damage are shown. Data are shown as mean ± SD of three independent experiments performed in duplicate. PARP expression was compared between RV-inoculated groups and their corresponding PBS-inoculated groups. The two-way ANOVA test was used, with a value of p****<0.0001 (B. C) Cells were stained with DAPI and analyzed by epifluorescence microscopy at the indicated times. Representative images from the cells analyzed in (A) are shown. The scale bar is 50 μm. (D) Quantitative analysis of images shown in (C) is expressed in terms of percentages of cells being positive for cell death proteins or Wt1-5 structural proteins. Cells positive for both viral proteins and cellular proteins are denoted as (+/+), while cells negative for both viral proteins and cellular proteins are denoted as (-/-). Data of three independent experiments performed in duplicate are shown as mean percentages ± standard deviation (SD). The Mann-Whitney U-test was used with a value of p*<0.05. (E) Western blot analysis of apoptosis-associated proteins pro-caspase-3 and pro-caspase-9 and cleaved caspase-9 and cleaved caspase-3 in rotavirus-infected gastric cancer tissues (12 h.p.i, MOI 0.8) was performed. Non-infected cells were used as a control. (F) The apoptosis and infection were evaluated using anti-Bcl-2, anti-BID, anti-PARP, and anti-RV staining in gastric cancer cells inoculated with Wt1-5 (MOI 0.2-12 h.p.i). Fluorescence was analyzed by flow cytometry. (G) Gastric tumors and adjacent non-cancerous tissues were infected with rotavirus (MOI 0.8) for 12, 24, or 60 h. Microphotographs of immunohistochemical staining of p53 protein with nuclear or predominantly nuclear staining (revealed with diaminobenzidine, seen as brown in the positive nuclei, with hematoxylin contrast giving a blue color to the negative nuclei) and (H) expression of Bcl-2 in the cytoplasm are shown. Original magnifications include x10, x20, x40 and x60, and black arrows show tumor cells. (I) The expression of p53 and Bcl-2 was quantified by the IHC Optical Density Score as the number of protein-positive cells using the HRP immunochemistry assay. Data are shown as mean ± SD of three independent experiments performed in duplicate. RV-inoculated groups were compared with the corresponding PBS-inoculated groups. The two-way ANOVA test was used, with a value of p****<0.0001. (J) Representative microphotographs of nuclear condensation and nuclear budding as the events inducing apoptosis-mediated cell death assessed at 48 h.p.i, MOI 0.8. Arrows are showing micronuclei, degenerated nuclei, and fragmented apoptotic nuclei in infected gastric cancer cells. (Scale bar = 50 μm).

To assess apoptosis induced by RV Wt1-5, flow cytometry was used to analyze the expression of pro-apoptotic proteins, including cleaved PARP-1, cytochrome-C, BAX, BID, cleaved caspase-3, cleaved caspase-9, as well as the anti-apoptotic protein Bcl-2 in gastric tumor cells and non-tumor cells inoculated with RV at different MOIs (MOI 0.2 or 0.8) after 12 hours of incubation. Cells inoculated with PBS were used as a control to evaluate marker expression. Our findings showed an increase in the expression of apoptotic proteins in gastric adenocarcinomas of the diffuse and intestinal-subtypes, as well as in the NCI-N87 cell line when inoculated with rotavirus, compared to uninfected cells. Moreover, the expression of PARP cleavage was dose-dependent in gastric cancer tissues. Interestingly, the expression of PARP cleavage in the adjacent non-tumor tissue decreased significantly only upon infection with RV (Fig 5A). This result is consistent with previous studies that have shown that in RV-infected cells, the RV protein NSP1 activates the PI3K/Akt pathway to prevent premature apoptosis associated with increased expression of the X-linked inhibitor of apoptosis (XIAP) and delayed cleavage of caspase 3 and poly (ADP-ribose) polymerase (PARP) [41]. Fig 5B and 5C show representative epifluorescence images of apoptotic proteins in gastric cancer cells inoculated with PBS or 12 h after RV infection (MOI 0.8). The association between RV Wt1-5 infection and the expression of cell death markers was evident when evaluating the percentages of cells that were positive or negative for the cell death proteins and the viral structural proteins. This analysis showed that cells were simultaneously positive for viral structural proteins and BAX, Casp-9, PARP-1, Cyt-C or BID (Fig 5D). Therefore, RV Wt1-5 infection can actively induce intrinsic apoptotic signaling through the proteins evaluated. Western blot was then used to evaluate the involvement of caspases 9 and 3 during rotavirus-induced cell death, as markers of early and late intrinsic apoptosis, respectively. As shown in Fig 5E, an increase in caspase-9 and caspase-3 activation was evident in RV-infected gastric cancer cells (MOI 0.8, 12 h.p.i) compared to the control group. In addition, the co-expression of Bcl-2 (16%), BID (12%), or PARP (18%) with the RV Wt1-5 (MOI 0.2) was determined by flow cytometry in gastric tumor cells 12 hours after infection (Fig 5F). The finding was that at 12 hours after infection, the expression of Bcl-2 and BAX was similar, but there was an increase in the expression of PARP in infected cells. In summary, these results suggest that RV Wt1-5 induces caspase-dependent or caspase-independent apoptosis in human GC cells at 12 h.p.i.

p53 is activated in response to several stress signals, such as viral infection, resulting in the inhibition of tumor cell growth, apoptosis, or necrosis by opening the mitochondrial permeability transition pore [42, 43]. Immunohistochemistry of tumor sections for p53 revealed a significantly increased p53 staining (>65%) in RV-treated tumors, whereas adjacent normal tissues did not show any effect over 24 h of infection (Fig 5G). Similarly, significant differences (p*<0.05) were found when quantifying p53 expression according to the IHC Optical Density Score in diffuse-subtype tumors infected with Wt1-5 (MOI 0.8) after 24 hours and in intestinal-subtype tumors after 12 hours when compared to the control, providing more information on the dose- and time-dependent selectivity and antitumor potential of RV (Fig 5I). Moreover, we evaluated the expression of the Bcl-2 protein, which inhibits the mitochondrial pathway of apoptosis. Its higher expression changes the balance between pro- and anti-apoptotic factors towards cell survival [44]. The expression levels of Bcl-2 were determined by immunohistochemistry in gastric cancer tissues infected or not with RV. Negative expression of Bcl-2 was found in tumors, and positive expression (65%) was found in adjacent non-tumor tissue, mainly in immune cells. A significant increase in the expression of Bcl-2 was observed 24 h.p.i with RV in gastric tumor tissue. However, it is important to note that in these tissues, Bcl-2 was mainly expressed in lymphocytes and endothelial cells (Fig 5H and 5I). Likewise, nuclear staining of infected tumor tissue showed pyknosis and karyorrhexis due to the cytopathic effect induced after rotaviral infection (Fig 5J).

**Fig 6:**
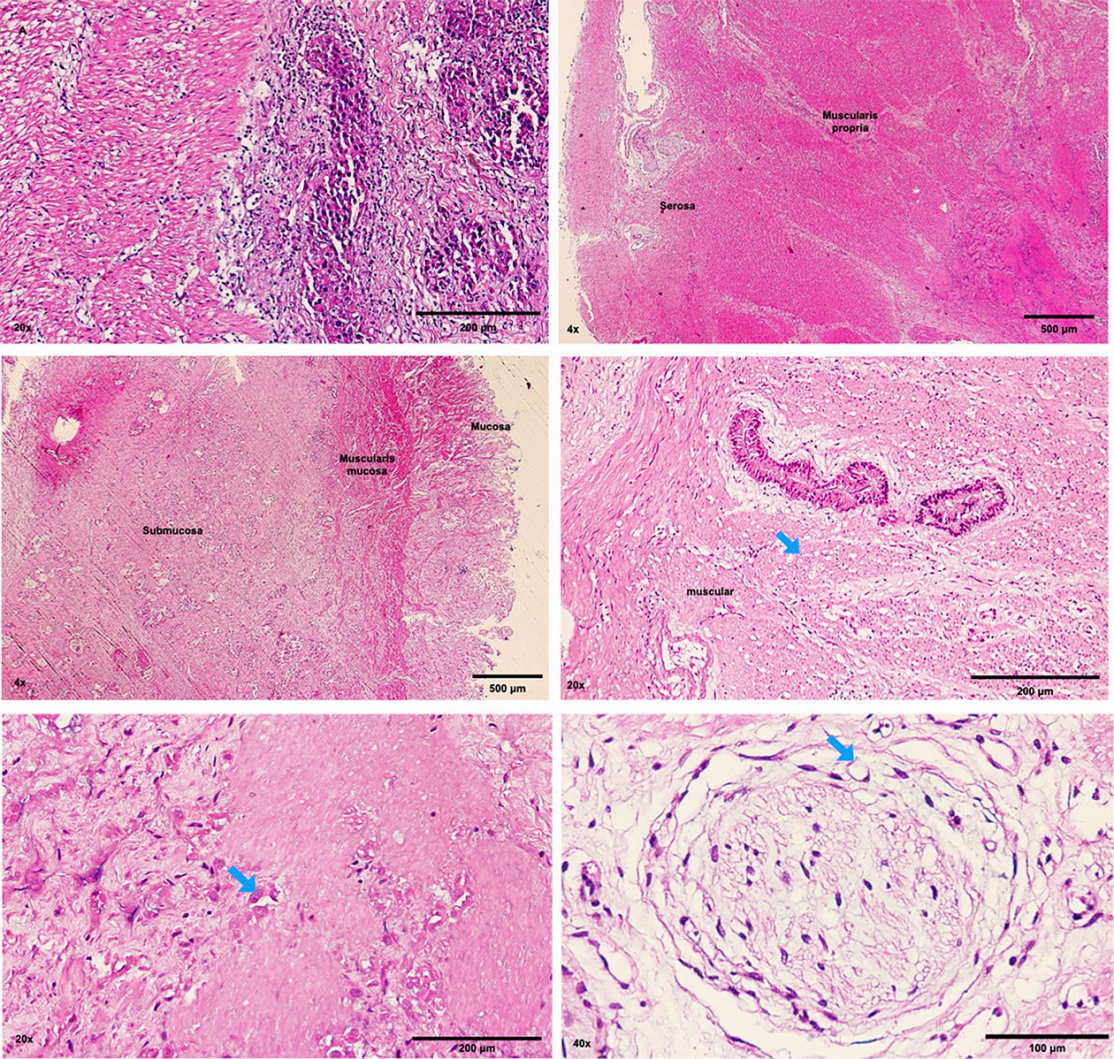
Gastric adenocarcinoma tissues were processed for histological hematoxylin and eosin (H&E) staining. **(A)** Tumors from mock-treated tissue showed no signs of necrosis. **(B and C)** Tumors from Wt 1-5—treated (60 h.p.i MOI 0.8) had areas of necrosis of corresponding to layers in the stomach walls. **(D)** The blue arrows show necrosis of the smooth muscle, **(E)** tumor cell necrosis, and **(F)** signet ring cell necrosis.

One of the main challenges in viral therapy is achieving effective spread and oncolytic activity throughout the tumor mass. To better characterize the antitumor effects of RV Wt1-5, histological analysis of gastric tumors treated with RV or mock after 60 hours was carried out. Cytopathic effects were not observed in control tissues (Fig 6A). In tumors treated with RV, areas of generalized tissue necrosis that compromised the depth of the tumor (mucosa, submucosa, muscular and serous) were observed (Fig 6B and 6F).

### Tumor microenvironment: Rotaviral spread and immune cell infiltrate

**Fig 7:**
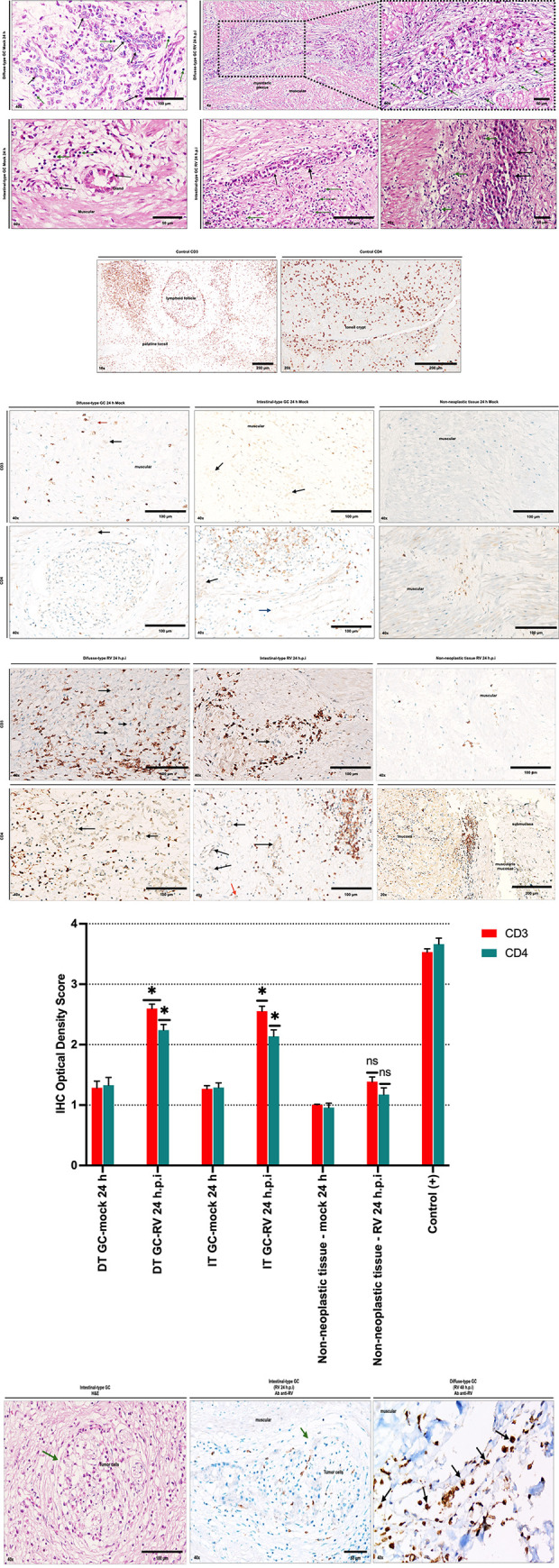
The immune microenvironment in cancer and adjacent non-cancerous tissues after infection with Wt 1-5 (24 or 48 h.p.i, MOI 0.8). **(A)** Representative photographs of H&E staining of gastric tumors treated with PBS and inoculated with RV Wt1-5 (24 h.p.i) are shown. **(B)** Palatine tonsil tissue was used as a positive control for immunocytochemical analysis. **(C)** Immunohistochemical analysis of lymphocytic infiltration in the peritumoral area and non-tumor tissues treated with PBS (24 h). The black arrows show tumor cell aggregates; the red arrow shows signet ring cells, and the blue arrow shows a blood vessel. **(D)** Immunohistochemical analysis of lymphocytic infiltration in the peritumoral area and non-tumor tissues after Wt1-5 infection (24 h.p.i, MOI 0.8). **(E)** The numbers of stained immune cells in tumors and adjacent-noncancerous tissues were quantified according to IHC Optical Density Score as the number of protein-positive cells using the HRP immunochemistry assay. **(F)** Samples were analyzed by H&E staining and rotavirus structural proteins staining. In H&E-stained sections, infiltrating cells appear in nervous tissue. Immunohistochemical reveals a strong cytoplasmic signal in invasive cancer and immune cells, including those for muscle invasion, vascular penetration, and nerve invasion.

Oncolytic virus therapy has been reported to reactivate local immune responses, as reflected by changes in the lymphocytic infiltrate, which are crucial for antitumor immunity [10, 45–47]. To determine whether RV Wt1-5 influences the T cell immune infiltrate, tumor and non-neoplastic tissue explants were inoculated with RV at an MOI of 0.8 or with PBS for 24 hours. Tissue samples were subjected to immunohistochemistry, and histological sections were analyzed by two pathologists. Representative photographs of H&E staining of gastric tumors treated with PBS and inoculated with RV Wt1-5 are shown in Fig 7A. Palatine tonsil tissue was used as a positive control for immunocytochemical analysis (Fig 7B). Fig 7C and 7D show representative images of the immunohistochemical staining of CD3 and CD4 lymphocytes in gastric tissues inoculated with or PBS or RV for 24 hours. IHC scores showed that diffuse-subtype and intestinal-subtype gastric adenocarcinomas infected with RV Wt1-5 (MOI 0.8) evidenced a significant increase in the population of CD3+ and CD4+ tumor-infiltrating T cells compared to tissues treated with PBS (p*<0.05). In non-neoplastic tissues, there was an increase in the population of CD4+ T lymphocytes after RV infection in lymphoid nodules of the muscularis mucosae tissue (Fig 7E). On the other hand, immunohistochemistry was used to evaluate the presence of rotavirus antigens in the explants inoculated with RV Wt1-5, highlighting the ability of the virus to spread within the tumor, compromising nerve structures and blood vessels, as observed with the presence of virus antigens within circulating immune cells. It has been described that specialized innate immune cells, such as DCs, are probably crucial in the control of RV replication in vivo [48] (Fig 7F).

## Discussion

The “hallmarks of cancer”, which include sustained proliferation, resistance to cell death, avoidance of growth suppressors, genome instability, stress from DNA damage, and escape from immune destruction, create an environment that is permissive for oncolytic viruses [49, 50]. The success of oncolytic viral therapy depends on coordinated mechanisms of action that involve penetration and dissemination in the tumor, tropism, and the virus´s mechanism of entry. The latter will depend on the repertoire, availability, and conformation of different receptors in neoplastic cells, as well as the indirect effects of the types of induced cell death that lead to reactivation of the immune system [51]. Moreover, the replication and spreading of OVs within human solid tumors are likely to be compromised by the restrictive properties of tumor architecture and microenvironment, such as extracellular matrix, hypoxia, high interstitial tumor pressure, and low pH) [52]. The first few days of OV infection and amplification within the tumor are critical because both the innate and adaptive immune systems eventually curtail the spread of the virus [37].

The ex vivo Infection model of live tumor tissue is crucial for a more precise study of the spreading capacity of rotavirus, its infectivity, cytopathic effects, and immune response dependent on tissue heterogeneity of the gastric tumor microenvironment (TME) [53]. This study demonstrates that after 12 hours, RV Wt1-5 had spread throughout the depth of intestinal and diffuse-subtype gastric adenocarcinomas, including structures such as nerve tissue and blood vessels, and that the tumor cells were significantly more infected than the adjacent non-tumor cells. These concentrations were sufficient to reach the threshold necessary to initiate an oncolytic effect. Another beneficial mechanism in OV therapy is the induction of innate and adaptive tumor-specific effector immunity, which exerts cytotoxicity against surviving cancer cells [54]. Interestingly, when evaluating the interactions of RV Wt1-5 with tumor-associated immune cells, a significant increase in lymphocytic infiltrate and viral antigens in phagocytic cells at the tumor microenvironment and in the lumen of the blood vessels was observed, suggesting that RV converted non-inflamed tumors into inflamed tumors, allowing activation of the host immune system. Rotaviruses are initially recognized by pattern recognition receptors (PRRs) in cells of the innate immune system such as macrophages and classical type I dendritic cells (cDC1) [55]. cDC1 excel at inducing CD8 T cells through cross-presentation, which is essential for optimal RV cytotoxicity, promoting strong adaptive immunity against RV infection [56]. Additionally, we show for the first time that RV is capable of infecting tumor vasculature, but the vascular endothelium in non-tumor tissues is resistant to virus infection. This is consistent with results found with other OVs that destroy tumor neovasculature, decreasing tumor progression and metastasis [37, 57].

The main objectives of oncolytic viral therapy are safety and efficacy, which require specificity towards tumor cells. In this regard, the formation of binding platforms between rotavirus proteins and the cell cytoskeleton has been shown, primarily through multiple proteins such as integrins (α2β1, αVβ3, αxV2) and heat shock proteins (Hsp90, Hsp70, Hsp60, Hsp40 and Hsc70). Thiol redox-dependent conformational changes and chaperone activity of disulfide isomerase proteins (PDIs) favor this interaction, allowing triple-layer particles (TLPs) to bind. The virus is then internalization into the host cell by receptor-mediated endocytosis or direct penetration of the cellular membrane [22, 23, 27, 28, 39, 40, 58]. Moreover, the overexpression and interactions of several heat shock proteins (Hsp70, Hsp90, Hsp60, Hsp40, Hsp27) [59–63], PDIs (Erp19, Erp57, TXNDC5, PDIA6, PDI) [64–67], integrins (αvβ5, α5β1, αvβ6, α2β1, α3β1, αvβ3) [68–70], and glycosphingolipid forming multiprotein complexes in the complex tumor microenvironment have been associated with cell malignancy and gastric cancer progression, favoring proliferation, survival, invasion, metastasis, and probably resistance to chemotherapy. These molecules are expressed at very low levels or not at all on the cell surface of normal cells, while gastric cancer cells express them at relatively high levels. In particular, Hsp70 has been detected in membrane rafts and shown to directly interact with the lipid phosphatidylserine in the plasma membranes of tumor cells [71]. Here, using flow cytometry and epifluorescence analysis, we have found that gastric tumor cells express moderate to high levels of heat shock proteins (Hsp40, Hsp60, Hsp70, Hsp90, Hsc70), integrins (β1, β2, and β3), and protein disulfide isomerase (PDI and ERp57) on their surface, while all the proteins evaluated were expressed at low levels in adjacent non-tumor tissue. Pre-incubation of gastric tumor cells with antibodies directed against these proteins resulted in decreased rotavirus infection, validating their importance for the binding and entry of the rotavirus into tumor cells, as previously reported [17, 20]. Interestingly, the gastric adenocarcinoma with the lowest expression of coreceptor proteins had the lowest percentage of infection at 12 h.p.i.

We aimed to determine the oncolytic capacity of rotavirus in tumors. Firstly, we demonstrated that RV Wt1-5 efficiently replicated in neoplastic cells by showing that the supernatant of rotavirus-infected tumor cells collected 12 hours after infection could infect the gastric cancer cell line NCI-N87. The direct cytopathic effects of RV Wt1-5 also include the possibility of inducing apoptosis in infected gastric cells. The rotavirus non-structural protein NSP4 induces apoptosis through ER stress in tumor cells by altering the permeability of the ER membrane, which causes an increase in the cytosolic concentration of Ca^2+^ [72, 73]. Therefore, we investigated the effect of rotavirus Wt1-5 infection on the expression of some apoptotic cell death markers. The evaluation of the ability to induce the expression of apoptotic proteins, including caspase 3, caspase 9, PARP, cytochrome C, BAX, BID, and Bcl-2, in gastric tumor tissue after 12 hours of RV infection showed early morphological changes associated with apoptosis, such as chromatin marginalization, nuclear condensation, and fragmentation. These results suggest that the infection leads in part to apoptotic death of neoplastic cells when compared with adjacent non-tumor tissue. Interestingly, increasing the MOI resulted in increased expression of apoptotic proteins. The expression levels of caspase-9 and associated proteins in the intrinsic apoptosis pathway are response markers used to predict therapeutic effect in cancer [74].

It is understood that the tumor suppressor p53 acts to integrate multiple stress signals into a series of diverse antiproliferative responses, one of the most important functions being its ability to induce changes in REDOX metabolism, leading to an increase in reactive oxygen species (ROS) and promoting caspase-independent cell death [75]. We examined the possible participation of the p53 protein in the cytotoxicity associated with infection and found significant time-dependent increases in p53. This result is consistent with what was observed during the initial stages of rotavirus infection, where the non-structural protein NSP1 degrades p53, inhibiting apoptosis. However, during late viral infection, the NSP1-p53 interaction is reduced, leading to the restoration of p53 levels and initiation of resulting pro-apoptotic signaling [76].

In addition, histological sections of infected tumors after 60 hours revealed areas of necrosis that compromised the entire thickness of the tumor. Moreover, from 12 hours post-infection, cells positive for viral antigens and negative for DAPI staining were observed, indicating the rupture and destruction of cellular DNA (Fig 2A). This mechanism of cell death, known as oncosis, has been associated with alterations in the transport and metabolism of calcium, leading to osmotic rupture, as previously reported in MA104 and Sp2 /0-Ag14 cancer cells [18, 77].

Our findings indicate that the rotaviral isolate spreads, replicates, and causes the death of gastric tumor cells through different mechanisms, including apoptosis initiated intrinsically in the early stages of infection and later by different death mechanisms, such as oncosis, demonstrating its antitumor efficacy. Therefore, it is feasible that caspase-dependent or independent cell death not only facilitates the release and spread of rotavirus but may also contribute to tissue necrosis. Additionally, it is essential to evaluate the interaction of oncolytic viruses with the tumor vasculature, considering their intratumoral or systemic application [37]. In this study, we observed an increase in the presence of viral antigens within circulating cells in the blood vessels of gastric tumor tissue compared to the vasculature of non-tumor tissue.

In conclusion, our findings in this preclinical model of oncolytic viral therapy demonstrate the capacity of RV Wt1-5 for tumor spread. Tumor tropism is dependent on the overexpression of receptors, such as Hsps, integrins, and PDIs, in the gastric tumor microenvironment. The main requirement for oncolytic viruses is the stimulation of an adaptive T-cell response against tumor-associated antigens. T-cell stimulation is induced by antigen-presenting cells, especially dendritic cells or macrophages. Our ex vivo explant model suggests exposure of immune cells to RV and a marked increase in lymphocytic infiltrate following infection. These findings suggest that the rotavirus isolate may be a potential oncolytic virus by simulating the lytic capacity in the living tumor tissue model.

## Supporting information

S1 File (DOCX)

S1 Text (TXT)

S2 Text (TXT)

S3 Text (TXT)
