# Supplementary material for: Preclinical evaluation of oncolytic potential human rotavirus Wt 1-5 in gastric adenocarcinoma
Source: PLoS One. 2023 May 15;18(5):e0285543. doi: 10.1371/journal.pone.0285543 (PMC10184912; doi:10.1371/journal.pone.0285543)
Supplement: S1 File — (DOCX) [file pone.0285543.s001.docx]

Resultados Dr Henry Abril 28 de 2022

. ranksum ck7 , by( ck7di )

Two-sample Wilcoxon rank-sum (Mann-Whitney) test

ck7di | obs rank sum expected

-------------+---------------------------------

a | 10 215 135

b | 16 136 216

-------------+---------------------------------

combined | 26 351 351

unadjusted variance 360.00

adjustment for ties -88.62

----------

adjusted variance 271.38

Ho: ck7(ck7di==a) = ck7(ck7di==b)

z = 4.856

Prob > |z| = 0.0000

. 0.05

0.05 is not a valid command name

r(199);

. drop*

. *(2 variables, 15 observations pasted into data editor)

. ranksum ck7 , by( ck7di )

Two-sample Wilcoxon rank-sum (Mann-Whitney) test

ck7di | obs rank sum expected

-------------+---------------------------------

a | 10 105 80

b | 5 15 40

-------------+---------------------------------

combined | 15 120 120

unadjusted variance 66.67

adjustment for ties -7.14

----------

adjusted variance 59.52

Ho: ck7(ck7di==a) = ck7(ck7di==b)

z = 3.240

Prob > |z| = 0.0012

. * Este valor p es menor a 0.05, por lo tanto es significativo. esto es que los v

> alores son diferentes al valor del grupo control.

. *(2 variables, 15 observations pasted into data editor)

. ranksum cea , by( ceadi )

Two-sample Wilcoxon rank-sum (Mann-Whitney) test

ceadi | obs rank sum expected

-------------+---------------------------------

a | 10 105 80

b | 5 15 40

-------------+---------------------------------

combined | 15 120 120

unadjusted variance 66.67

adjustment for ties -7.14

----------

adjusted variance 59.52

Ho: cea(ceadi==a) = cea(ceadi==b)

z = 3.240

Prob > |z| = 0.0012

. * Este valor p es menor a 0.05, por lo tanto es significativo. esto es que los v

> alores son diferentes al valor del grupo control.

. *(4 variables, 15 observations pasted into data editor)

(variable named "hpi" already exists; using name "var7")

(variable named "hpiinf" already exists; using name "var8")

. drop hpi hpiinf var7 var8

. *(4 variables, 15 observations pasted into data editor)

. ranksum hpi12 , by( hpi12inf )

Two-sample Wilcoxon rank-sum (Mann-Whitney) test

hpi12inf | obs rank sum expected

-------------+---------------------------------

a | 10 105 80

b | 5 15 40

-------------+---------------------------------

combined | 15 120 120

unadjusted variance 66.67

adjustment for ties -7.14

----------

adjusted variance 59.52

Ho: hpi12(hpi12inf==a) = hpi12(hpi12inf==b)

z = 3.240

Prob > |z| = 0.0012

. ranksum hpi24 , by( hpi24inf )

Two-sample Wilcoxon rank-sum (Mann-Whitney) test

hpi24inf | obs rank sum expected

-------------+---------------------------------

a | 10 105 80

b | 5 15 40

-------------+---------------------------------

combined | 15 120 120

unadjusted variance 66.67

adjustment for ties -7.14

----------

adjusted variance 59.52

Ho: hpi24(hpi24inf==a) = hpi24(hpi24inf==b)

z = 3.240

Prob > |z| = 0.0012

. *(2 variables, 10 observations pasted into data editor)

. ranksum tumor , by( tumordi )

Two-sample Wilcoxon rank-sum (Mann-Whitney) test

tumordi | obs rank sum expected

-------------+---------------------------------

a | 5 15 27.5

b | 5 40 27.5

-------------+---------------------------------

combined | 10 55 55

unadjusted variance 22.92

adjustment for ties -2.78

----------

adjusted variance 20.14

Ho: tumor(tumordi==a) = tumor(tumordi==b)

z = -2.785

Prob > |z| = 0.0053

. * Este valor p es menor a 0.05, por lo tanto es significativo. esto es que los v

> alores son diferentes al valor del grupo control.

. *(2 variables, 8 observations pasted into data editor)

. ranksum tumordi , by( tumordifu )

no observations

r(2000);

. replace tumordifu = 40 in 2

(1 real change made)

. replace tumordifu = 40 in 3

(1 real change made)

. replace tumordifu = 40 in 4

(1 real change made)

. ranksum tumordi , by( tumordifu )

no observations

r(2000);

. drop tumordi tumordifuso

. *(2 variables, 8 observations pasted into data editor)

(variable named "tumordifu" already exists; using name "var11")

. ranksum var11 , by( tumordifu )

more than 2 groups found, only 2 allowed

r(499);

. drop var11 tumordifu tumordifuso

. *(2 variables, 8 observations pasted into data editor)

. ranksum difuso , by( difusotu )

Two-sample Wilcoxon rank-sum (Mann-Whitney) test

difusotu | obs rank sum expected

-------------+---------------------------------

a | 4 10 18

b | 4 26 18

-------------+---------------------------------

combined | 8 36 36

unadjusted variance 12.00

adjustment for ties -1.43

----------

adjusted variance 10.57

Ho: difuso(difusotu==a) = difuso(difusotu==b)

z = -2.460

Prob > |z| = 0.0139

. * Este valor p es menor a 0.05, por lo tanto es significativo. esto es que los v

> alores son diferentes al valor del grupo control.

. *(2 variables, 15 observations pasted into data editor)

. ranksum fluore , by( fluoredi )

Two-sample Wilcoxon rank-sum (Mann-Whitney) test

fluoredi | obs rank sum expected

-------------+---------------------------------

a | 10 105 80

b | 5 15 40

-------------+---------------------------------

combined | 15 120 120

unadjusted variance 66.67

adjustment for ties -7.14

----------

adjusted variance 59.52

Ho: fluore(fluoredi==a) = fluore(fluoredi==b)

z = 3.240

Prob > |z| = 0.0012

. * Este valor p es menor a 0.05, por lo tanto es significativo. esto es que los v

> alores son diferentes al valor del grupo control.

. *(2 variables, 10 observations pasted into data editor)

. ranksum celtej , by( celtejdi )

Two-sample Wilcoxon rank-sum (Mann-Whitney) test

celtejdi | obs rank sum expected

-------------+---------------------------------

a | 5 15 27.5

b | 5 40 27.5

-------------+---------------------------------

combined | 10 55 55

unadjusted variance 22.92

adjustment for ties -5.56

----------

adjusted variance 17.36

Ho: celtej(celtejdi==a) = celtej(celtejdi==b)

z = -3.000

Prob > |z| = 0.0027

. * Este valor p es menor a 0.05, por lo tanto es significativo. esto es que los v

> alores son diferentes al valor del grupo control.

. *(10 variables, 10 observations pasted into data editor)

. ranksum hsp40 , by( marcadores )

Two-sample Wilcoxon rank-sum (Mann-Whitney) test

marcadores | obs rank sum expected

-------------+---------------------------------

a | 5 30 27.5

b | 5 25 27.5

-------------+---------------------------------

combined | 10 55 55

unadjusted variance 22.92

adjustment for ties -2.92

----------

adjusted variance 20.00

Ho: hsp40(marcad~s==a) = hsp40(marcad~s==b)

z = 0.559

Prob > |z| = 0.5762

. * valor p mayor a 0.05. no hay diferencias con el grupo control

. ranksum hsp60 , by( marcadores )

Two-sample Wilcoxon rank-sum (Mann-Whitney) test

marcadores | obs rank sum expected

-------------+---------------------------------

a | 5 25 27.5

b | 5 30 27.5

-------------+---------------------------------

combined | 10 55 55

unadjusted variance 22.92

adjustment for ties -2.78

----------

adjusted variance 20.14

Ho: hsp60(marcad~s==a) = hsp60(marcad~s==b)

z = -0.557

Prob > |z| = 0.5775

. ranksum hsp70 , by( marcadores )

Two-sample Wilcoxon rank-sum (Mann-Whitney) test

marcadores | obs rank sum expected

-------------+---------------------------------

a | 5 30 27.5

b | 5 25 27.5

-------------+---------------------------------

combined | 10 55 55

unadjusted variance 22.92

adjustment for ties -2.92

----------

adjusted variance 20.00

Ho: hsp70(marcad~s==a) = hsp70(marcad~s==b)

z = 0.559

Prob > |z| = 0.5762

. ranksum hsp90 , by( marcadores )

Two-sample Wilcoxon rank-sum (Mann-Whitney) test

marcadores | obs rank sum expected

-------------+---------------------------------

a | 5 35 27.5

b | 5 20 27.5

-------------+---------------------------------

combined | 10 55 55

unadjusted variance 22.92

adjustment for ties -2.78

----------

adjusted variance 20.14

Ho: hsp90(marcad~s==a) = hsp90(marcad~s==b)

z = 1.671

Prob > |z| = 0.0947

. ranksum hsc70 , by( marcadores )

Two-sample Wilcoxon rank-sum (Mann-Whitney) test

marcadores | obs rank sum expected

-------------+---------------------------------

a | 5 35 27.5

b | 5 20 27.5

-------------+---------------------------------

combined | 10 55 55

unadjusted variance 22.92

adjustment for ties -2.78

----------

adjusted variance 20.14

Ho: hsc70(marcad~s==a) = hsc70(marcad~s==b)

z = 1.671

Prob > |z| = 0.0947

. ranksum beta1 , by( marcadores )

Two-sample Wilcoxon rank-sum (Mann-Whitney) test

marcadores | obs rank sum expected

-------------+---------------------------------

a | 5 30 27.5

b | 5 25 27.5

-------------+---------------------------------

combined | 10 55 55

unadjusted variance 22.92

adjustment for ties -2.78

----------

adjusted variance 20.14

Ho: beta1(marcad~s==a) = beta1(marcad~s==b)

z = 0.557

Prob > |z| = 0.5775

. ranksum beta3 , by( marcadores )

variable beta3 not found

r(111);

. ranksum beat3 , by( marcadores )

Two-sample Wilcoxon rank-sum (Mann-Whitney) test

marcadores | obs rank sum expected

-------------+---------------------------------

a | 5 35 27.5

b | 5 20 27.5

-------------+---------------------------------

combined | 10 55 55

unadjusted variance 22.92

adjustment for ties -2.78

----------

adjusted variance 20.14

Ho: beat3(marcad~s==a) = beat3(marcad~s==b)

z = 1.671

Prob > |z| = 0.0947

. ranksum pdi , by( marcadores )

Two-sample Wilcoxon rank-sum (Mann-Whitney) test

marcadores | obs rank sum expected

-------------+---------------------------------

a | 5 30 27.5

b | 5 25 27.5

-------------+---------------------------------

combined | 10 55 55

unadjusted variance 22.92

adjustment for ties -2.78

----------

adjusted variance 20.14

Ho: pdi(marcad~s==a) = pdi(marcad~s==b)

z = 0.557

Prob > |z| = 0.5775

. ranksum erp57 , by( marcadores )

Two-sample Wilcoxon rank-sum (Mann-Whitney) test

marcadores | obs rank sum expected

-------------+---------------------------------

a | 5 30 27.5

b | 5 25 27.5

-------------+---------------------------------

combined | 10 55 55

unadjusted variance 22.92

adjustment for ties -2.92

----------

adjusted variance 20.00

Ho: erp57(marcad~s==a) = erp57(marcad~s==b)

z = 0.559

Prob > |z| = 0.5762

. drop hsp40 hsp60 hsp70 hsp90 hsc70 beta1 beat3 pdi marcadores

. *(10 variables, 10 observations pasted into data editor)

(variable named "erp57" already exists; using name "var25")

. ranksum hsp60 , by( marcadores )

Two-sample Wilcoxon rank-sum (Mann-Whitney) test

marcadores | obs rank sum expected

-------------+---------------------------------

a | 5 40 27.5

b | 5 15 27.5

-------------+---------------------------------

combined | 10 55 55

unadjusted variance 22.92

adjustment for ties -5.56

----------

adjusted variance 17.36

Ho: hsp60(marcad~s==a) = hsp60(marcad~s==b)

z = 3.000

Prob > |z| = 0.0027

. ranksum hsp70 , by( marcadores )

Two-sample Wilcoxon rank-sum (Mann-Whitney) test

marcadores | obs rank sum expected

-------------+---------------------------------

a | 5 40 27.5

b | 5 15 27.5

-------------+---------------------------------

combined | 10 55 55

unadjusted variance 22.92

adjustment for ties -5.56

----------

adjusted variance 17.36

Ho: hsp70(marcad~s==a) = hsp70(marcad~s==b)

z = 3.000

Prob > |z| = 0.0027

. ranksum hsp90 , by( marcadores )

Two-sample Wilcoxon rank-sum (Mann-Whitney) test

marcadores | obs rank sum expected

-------------+---------------------------------

a | 5 40 27.5

b | 5 15 27.5

-------------+---------------------------------

combined | 10 55 55

unadjusted variance 22.92

adjustment for ties -5.56

----------

adjusted variance 17.36

Ho: hsp90(marcad~s==a) = hsp90(marcad~s==b)

z = 3.000

Prob > |z| = 0.0027

. ranksum hsc70 , by( marcadores )

Two-sample Wilcoxon rank-sum (Mann-Whitney) test

marcadores | obs rank sum expected

-------------+---------------------------------

a | 5 40 27.5

b | 5 15 27.5

-------------+---------------------------------

combined | 10 55 55

unadjusted variance 22.92

adjustment for ties -5.56

----------

adjusted variance 17.36

Ho: hsc70(marcad~s==a) = hsc70(marcad~s==b)

z = 3.000

Prob > |z| = 0.0027

. ranksum beta1 , by( marcadores )

Two-sample Wilcoxon rank-sum (Mann-Whitney) test

marcadores | obs rank sum expected

-------------+---------------------------------

a | 5 40 27.5

b | 5 15 27.5

-------------+---------------------------------

combined | 10 55 55

unadjusted variance 22.92

adjustment for ties -5.56

----------

adjusted variance 17.36

Ho: beta1(marcad~s==a) = beta1(marcad~s==b)

z = 3.000

Prob > |z| = 0.0027

. ranksum beat3 , by( marcadores )

Two-sample Wilcoxon rank-sum (Mann-Whitney) test

marcadores | obs rank sum expected

-------------+---------------------------------

a | 5 40 27.5

b | 5 15 27.5

-------------+---------------------------------

combined | 10 55 55

unadjusted variance 22.92

adjustment for ties -5.56

----------

adjusted variance 17.36

Ho: beat3(marcad~s==a) = beat3(marcad~s==b)

z = 3.000

Prob > |z| = 0.0027

. ranksum pdi , by( marcadores )

Two-sample Wilcoxon rank-sum (Mann-Whitney) test

marcadores | obs rank sum expected

-------------+---------------------------------

a | 5 40 27.5

b | 5 15 27.5

-------------+---------------------------------

combined | 10 55 55

unadjusted variance 22.92

adjustment for ties -5.56

----------

adjusted variance 17.36

Ho: pdi(marcad~s==a) = pdi(marcad~s==b)

z = 3.000

Prob > |z| = 0.0027

. ranksum var25 , by( marcadores )

Two-sample Wilcoxon rank-sum (Mann-Whitney) test

marcadores | obs rank sum expected

-------------+---------------------------------

a | 5 40 27.5

b | 5 15 27.5

-------------+---------------------------------

combined | 10 55 55

unadjusted variance 22.92

adjustment for ties -5.56

----------

adjusted variance 17.36

Ho: var25(marcad~s==a) = var25(marcad~s==b)

z = 3.000

Prob > |z| = 0.0027

. *(2 variables, 42 observations pasted into data editor)

. ranksum membranamembdi , by( var28 )

Two-sample Wilcoxon rank-sum (Mann-Whitney) test

var28 | obs rank sum expected

-------------+---------------------------------

a | 21 672 451.5

b | 21 231 451.5

-------------+---------------------------------

combined | 42 903 903

unadjusted variance 1580.25

adjustment for ties -55.57

----------

adjusted variance 1524.68

Ho: membra~i(var28==a) = membra~i(var28==b)

z = 5.647

Prob > |z| = 0.0000

. *(7 variables, 10 observations pasted into data editor)

. ranksum antihsp90 , by( marcadores )

Two-sample Wilcoxon rank-sum (Mann-Whitney) test

marcadores | obs rank sum expected

-------------+---------------------------------

a | 5 15 27.5

b | 5 40 27.5

-------------+---------------------------------

combined | 10 55 55

unadjusted variance 22.92

adjustment for ties -5.56

----------

adjusted variance 17.36

Ho: antih~90(marcad~s==a) = antih~90(marcad~s==b)

z = -3.000

Prob > |z| = 0.0027

. ranksum antihsc70 , by( marcadores )

Two-sample Wilcoxon rank-sum (Mann-Whitney) test

marcadores | obs rank sum expected

-------------+---------------------------------

a | 5 15 27.5

b | 5 40 27.5

-------------+---------------------------------

combined | 10 55 55

unadjusted variance 22.92

adjustment for ties -5.56

----------

adjusted variance 17.36

Ho: anti~c70(marcad~s==a) = anti~c70(marcad~s==b)

z = -3.000

Prob > |z| = 0.0027

. ranksum antiαVβ3 , by( marcadores )

Two-sample Wilcoxon rank-sum (Mann-Whitney) test

marcadores | obs rank sum expected

-------------+---------------------------------

a | 5 15 27.5

b | 5 40 27.5

-------------+---------------------------------

combined | 10 55 55

unadjusted variance 22.92

adjustment for ties -5.56

----------

adjusted variance 17.36

Ho: antiαVβ3(marcad~s==a) = antiαVβ3(marcad~s==b)

z = -3.000

Prob > |z| = 0.0027

. ranksum antihsp40 , by( marcadores )

Two-sample Wilcoxon rank-sum (Mann-Whitney) test

marcadores | obs rank sum expected

-------------+---------------------------------

a | 5 15 27.5

b | 5 40 27.5

-------------+---------------------------------

combined | 10 55 55

unadjusted variance 22.92

adjustment for ties -5.56

----------

adjusted variance 17.36

Ho: antih~40(marcad~s==a) = antih~40(marcad~s==b)

z = -3.000

Prob > |z| = 0.0027

. ranksum antihsp60 , by( marcadores )

Two-sample Wilcoxon rank-sum (Mann-Whitney) test

marcadores | obs rank sum expected

-------------+---------------------------------

a | 5 15 27.5

b | 5 40 27.5

-------------+---------------------------------

combined | 10 55 55

unadjusted variance 22.92

adjustment for ties -5.56

----------

adjusted variance 17.36

Ho: antih~60(marcad~s==a) = antih~60(marcad~s==b)

z = -3.000

Prob > |z| = 0.0027

. ranksum antihsp70 , by( marcadores )

Two-sample Wilcoxon rank-sum (Mann-Whitney) test

marcadores | obs rank sum expected

-------------+---------------------------------

a | 5 15 27.5

b | 5 40 27.5

-------------+---------------------------------

combined | 10 55 55

unadjusted variance 22.92

adjustment for ties -5.56

----------

adjusted variance 17.36

Ho: anti~p70(marcad~s==a) = anti~p70(marcad~s==b)

z = -3.000

Prob > |z| = 0.0027

. ranksum antipdi , by( marcadores )

Two-sample Wilcoxon rank-sum (Mann-Whitney) test

marcadores | obs rank sum expected

-------------+---------------------------------

a | 5 15 27.5

b | 5 40 27.5

-------------+---------------------------------

combined | 10 55 55

unadjusted variance 22.92

adjustment for ties -5.56

----------

adjusted variance 17.36

Ho: antipdi(marcad~s==a) = antipdi(marcad~s==b)

z = -3.000

Prob > |z| = 0.0027

. *(1 variable, 10 observations pasted into data editor)

. ranksum parp , by( marcadores )

Two-sample Wilcoxon rank-sum (Mann-Whitney) test

marcadores | obs rank sum expected

-------------+---------------------------------

a | 5 15 27.5

b | 5 40 27.5

-------------+---------------------------------

combined | 10 55 55

unadjusted variance 22.92

adjustment for ties -5.56

----------

adjusted variance 17.36

Ho: parp(marcad~s==a) = parp(marcad~s==b)

z = -3.000

Prob > |z| = 0.0027

. *(2 variables, 15 observations pasted into data editor)

. kwallis casp3 , by( marcadores3 )

Kruskal-Wallis equality-of-populations rank test

+---------------------------+

| marcad~3 | Obs | Rank Sum |

|----------+-----+----------|

| a | 5 | 40.00 |

| b | 5 | 15.00 |

| c | 5 | 65.00 |

+---------------------------+

chi-squared = 12.500 with 2 d.f.

probability = 0.0019

chi-squared with ties = 14.000 with 2 d.f.

probability = 0.0009

. * Este valor p es menor a 0.05, por lo tanto es significativo. esto es que los v

> alores son diferentes al valor del grupo control.

. * la prueba anterior fue KWallis

. dunntest casp3 , by( marcadores3 ) ma(bonferroni)

Warning: by() values are unlabeled, option nolabel implicit

Kruskal-Wallis equality-of-populations rank test

+---------------------------+

| marcad~3 | Obs | Rank Sum |

|----------+-----+----------|

| a | 5 | 40.00 |

| b | 5 | 15.00 |

| c | 5 | 65.00 |

+---------------------------+

chi-squared = 12.500 with 2 d.f.

probability = 0.0019

chi-squared with ties = 14.000 with 2 d.f.

probability = 0.0009

type mismatch

r(109);

. dunntest casp3 , by( marcadores3 )

Warning: by() values are unlabeled, option nolabel implicit

Kruskal-Wallis equality-of-populations rank test

+---------------------------+

| marcad~3 | Obs | Rank Sum |

|----------+-----+----------|

| a | 5 | 40.00 |

| b | 5 | 15.00 |

| c | 5 | 65.00 |

+---------------------------+

chi-squared = 12.500 with 2 d.f.

probability = 0.0019

chi-squared with ties = 14.000 with 2 d.f.

probability = 0.0009

type mismatch

r(109);

. dunntest casp3, by( marcadores3 )

Warning: by() values are unlabeled, option nolabel implicit

Kruskal-Wallis equality-of-populations rank test

+---------------------------+

| marcad~3 | Obs | Rank Sum |

|----------+-----+----------|

| a | 5 | 40.00 |

| b | 5 | 15.00 |

| c | 5 | 65.00 |

+---------------------------+

chi-squared = 12.500 with 2 d.f.

probability = 0.0019

chi-squared with ties = 14.000 with 2 d.f.

probability = 0.0009

type mismatch

r(109);

. kwallis casp3 , by( marcadores3 )

Kruskal-Wallis equality-of-populations rank test

+---------------------------+

| marcad~3 | Obs | Rank Sum |

|----------+-----+----------|

| a | 5 | 40.00 |

| b | 5 | 15.00 |

| c | 5 | 65.00 |

+---------------------------+

chi-squared = 12.500 with 2 d.f.

probability = 0.0019

chi-squared with ties = 14.000 with 2 d.f.

probability = 0.0009

. dunntest casp3, by(marcadores3) ma(bonferroni)

Warning: by() values are unlabeled, option nolabel implicit

Kruskal-Wallis equality-of-populations rank test

+---------------------------+

| marcad~3 | Obs | Rank Sum |

|----------+-----+----------|

| a | 5 | 40.00 |

| b | 5 | 15.00 |

| c | 5 | 65.00 |

+---------------------------+

chi-squared = 12.500 with 2 d.f.

probability = 0.0019

chi-squared with ties = 14.000 with 2 d.f.

probability = 0.0009

type mismatch

r(109);

. *(1 variable, 10 observations pasted into data editor)

. ranksum nci , by( marcadores )

Two-sample Wilcoxon rank-sum (Mann-Whitney) test

marcadores | obs rank sum expected

-------------+---------------------------------

a | 5 15 27.5

b | 5 40 27.5

-------------+---------------------------------

combined | 10 55 55

unadjusted variance 22.92

adjustment for ties -5.56

----------

adjusted variance 17.36

Ho: nci(marcad~s==a) = nci(marcad~s==b)

z = -3.000

Prob > |z| = 0.0027

. *(1 variable, 10 observations pasted into data editor)

. ranksum difuso24 , by( marcadores )

Two-sample Wilcoxon rank-sum (Mann-Whitney) test

marcadores | obs rank sum expected

-------------+---------------------------------

a | 5 15 27.5

b | 5 40 27.5

-------------+---------------------------------

combined | 10 55 55

unadjusted variance 22.92

adjustment for ties -5.56

----------

adjusted variance 17.36

Ho: difuso24(marcad~s==a) = difuso24(marcad~s==b)

z = -3.000

Prob > |z| = 0.0027

. *(1 variable, 10 observations pasted into data editor)

. ranksum intestinal , by( marcadores )

Two-sample Wilcoxon rank-sum (Mann-Whitney) test

marcadores | obs rank sum expected

-------------+---------------------------------

a | 5 15 27.5

b | 5 40 27.5

-------------+---------------------------------

combined | 10 55 55

unadjusted variance 22.92

adjustment for ties -5.56

----------

adjusted variance 17.36

Ho: intest~l(marcad~s==a) = intest~l(marcad~s==b)

z = -3.000

Prob > |z| = 0.0027

. *(1 variable, 15 observations pasted into data editor)

. ranksum cd3 , by( marcadores )

Two-sample Wilcoxon rank-sum (Mann-Whitney) test

marcadores | obs rank sum expected

-------------+---------------------------------

a | 5 40 27.5

b | 5 15 27.5

-------------+---------------------------------

combined | 10 55 55

unadjusted variance 22.92

adjustment for ties -5.56

----------

adjusted variance 17.36

Ho: cd3(marcad~s==a) = cd3(marcad~s==b)

z = 3.000

Prob > |z| = 0.0027

. *(2 variables, 15 observations pasted into data editor)

(variable named "cd3" already exists; using name "var43")

. ranksum cd3 , by( marcadores3 )

more than 2 groups found, only 2 allowed

r(499);

. kwallis cd3 , by( marcadores3 )

Kruskal-Wallis equality-of-populations rank test

+---------------------------+

| marcad~3 | Obs | Rank Sum |

|----------+-----+----------|

| a | 5 | 65.00 |

| b | 5 | 40.00 |

| c | 5 | 15.00 |

+---------------------------+

chi-squared = 12.500 with 2 d.f.

probability = 0.0019

chi-squared with ties = 14.000 with 2 d.f.

probability = 0.0009

. kwallis var43 , by( marcadores3 )

Kruskal-Wallis equality-of-populations rank test

+---------------------------+

| marcad~3 | Obs | Rank Sum |

|----------+-----+----------|

| a | 5 | 65.00 |

| b | 5 | 40.00 |

| c | 5 | 15.00 |

+---------------------------+

chi-squared = 12.500 with 2 d.f.

probability = 0.0019

chi-squared with ties = 14.000 with 2 d.f.

probability = 0.0009

. *(1 variable, 10 observations pasted into data editor)

. ranksum cd3tumor , by( marcadores )

Two-sample Wilcoxon rank-sum (Mann-Whitney) test

marcadores | obs rank sum expected

-------------+---------------------------------

a | 5 15 27.5

b | 5 40 27.5

-------------+---------------------------------

combined | 10 55 55

unadjusted variance 22.92

adjustment for ties -5.56

----------

adjusted variance 17.36

Ho: cd3tumor(marcad~s==a) = cd3tumor(marcad~s==b)

z = -3.000

Prob > |z| = 0.0027

. *(10 variables, 25 observations pasted into data editor)

. kwallis hsp40kw , by( marcador5 )

Kruskal-Wallis equality-of-populations rank test

+---------------------------+

| marcad~5 | Obs | Rank Sum |

|----------+-----+----------|

| a | 5 | 15.00 |

| b | 5 | 65.00 |

| c | 5 | 115.00 |

| d | 5 | 90.00 |

| e | 5 | 40.00 |

+---------------------------+

chi-squared = 23.077 with 4 d.f.

probability = 0.0001

chi-squared with ties = 24.000 with 4 d.f.

probability = 0.0001

. dunntest hsp40kw , by( marcador5 ) ma(bonferroni)

Warning: by() values are unlabeled, option nolabel implicit

Kruskal-Wallis equality-of-populations rank test

+---------------------------+

| marcad~5 | Obs | Rank Sum |

|----------+-----+----------|

| a | 5 | 15.00 |

| b | 5 | 65.00 |

| c | 5 | 115.00 |

| d | 5 | 90.00 |

| e | 5 | 40.00 |

+---------------------------+

chi-squared = 23.077 with 4 d.f.

probability = 0.0001

chi-squared with ties = 24.000 with 4 d.f.

probability = 0.0001

type mismatch

r(109);

. kwallis hsp60kw , by( marcador5 )

Kruskal-Wallis equality-of-populations rank test

+---------------------------+

| marcad~5 | Obs | Rank Sum |

|----------+-----+----------|

| a | 5 | 40.00 |

| b | 5 | 65.00 |

| c | 5 | 115.00 |

| d | 5 | 90.00 |

| e | 5 | 15.00 |

+---------------------------+

chi-squared = 23.077 with 4 d.f.

probability = 0.0001

chi-squared with ties = 24.000 with 4 d.f.

probability = 0.0001

. kwallis hsp70kw , by( marcador5 )

Kruskal-Wallis equality-of-populations rank test

+---------------------------+

| marcad~5 | Obs | Rank Sum |

|----------+-----+----------|

| a | 5 | 15.00 |

| b | 5 | 65.00 |

| c | 5 | 115.00 |

| d | 5 | 90.00 |

| e | 5 | 40.00 |

+---------------------------+

chi-squared = 23.077 with 4 d.f.

probability = 0.0001

chi-squared with ties = 24.000 with 4 d.f.

probability = 0.0001

. kwallis hsp90kw , by( marcador5 )

Kruskal-Wallis equality-of-populations rank test

+---------------------------+

| marcad~5 | Obs | Rank Sum |

|----------+-----+----------|

| a | 5 | 65.00 |

| b | 5 | 40.00 |

| c | 5 | 115.00 |

| d | 5 | 90.00 |

| e | 5 | 15.00 |

+---------------------------+

chi-squared = 23.077 with 4 d.f.

probability = 0.0001

chi-squared with ties = 24.000 with 4 d.f.

probability = 0.0001

. kwallis hsc70kw , by( marcador5 )

Kruskal-Wallis equality-of-populations rank test

+---------------------------+

| marcad~5 | Obs | Rank Sum |

|----------+-----+----------|

| a | 5 | 40.00 |

| b | 5 | 90.00 |

| c | 5 | 115.00 |

| d | 5 | 65.00 |

| e | 5 | 15.00 |

+---------------------------+

chi-squared = 23.077 with 4 d.f.

probability = 0.0001

chi-squared with ties = 24.000 with 4 d.f.

probability = 0.0001

. kwallis β1kw , by( marcador5 )

Kruskal-Wallis equality-of-populations rank test

+---------------------------+

| marcad~5 | Obs | Rank Sum |

|----------+-----+----------|

| a | 5 | 40.00 |

| b | 5 | 65.00 |

| c | 5 | 115.00 |

| d | 5 | 90.00 |

| e | 5 | 15.00 |

+---------------------------+

chi-squared = 23.077 with 4 d.f.

probability = 0.0001

chi-squared with ties = 24.000 with 4 d.f.

probability = 0.0001

. kwallis αVβ3kw , by( marcador5 )

Kruskal-Wallis equality-of-populations rank test

+---------------------------+

| marcad~5 | Obs | Rank Sum |

|----------+-----+----------|

| a | 5 | 40.00 |

| b | 5 | 65.00 |

| c | 5 | 115.00 |

| d | 5 | 90.00 |

| e | 5 | 15.00 |

+---------------------------+

chi-squared = 23.077 with 4 d.f.

probability = 0.0001

chi-squared with ties = 24.000 with 4 d.f.

probability = 0.0001

. kwallis pdikw , by( marcador5 )

Kruskal-Wallis equality-of-populations rank test

+---------------------------+

| marcad~5 | Obs | Rank Sum |

|----------+-----+----------|

| a | 5 | 65.00 |

| b | 5 | 90.00 |

| c | 5 | 40.00 |

| d | 5 | 115.00 |

| e | 5 | 15.00 |

+---------------------------+

chi-squared = 23.077 with 4 d.f.

probability = 0.0001

chi-squared with ties = 24.000 with 4 d.f.

probability = 0.0001

. kwallis erp57kw , by( marcador5 )

Kruskal-Wallis equality-of-populations rank test

+---------------------------+

| marcad~5 | Obs | Rank Sum |

|----------+-----+----------|

| a | 5 | 65.00 |

| b | 5 | 40.00 |

| c | 5 | 115.00 |

| d | 5 | 90.00 |

| e | 5 | 15.00 |

+---------------------------+

chi-squared = 23.077 with 4 d.f.

probability = 0.0001

chi-squared with ties = 24.000 with 4 d.f.

probability = 0.0001
